# Investigating the role of drama-based strategies in addressing psychological trauma: a systematic review

**DOI:** 10.1080/20008066.2026.2739019

**Published:** 2026-10-20

**Authors:** Eliza Oliver, Lucinda Grummitt, Robyn Ewing, Louise Birrell

**Affiliations:** a The Matilda Centre for Research in Mental Health and Substance Use, University of Sydney, Sydney, Australia; b The CREATE Centre, University of Sydney, Sydney, Australia

**Keywords:** Trauma, PTSD, drama, dramatherapy, review, Trauma, TEPT, drama, dramaterapia, revisión

## Abstract

**Background:** Drama-based techniques have been increasingly implemented with trauma-exposed populations. However, the evidence base remains conceptually and methodologically heterogeneous, with substantial variation in how trauma is defined, how interventions are described, and which trauma-related outcomes are assessed.

**Objective:** This review aimed to synthesise empirical studies examining drama-based interventions for individuals exposed to trauma. The review describes the characteristics of drama-based interventions, the types of trauma addressed, the pattern and range of trauma-related psychological outcomes assessed, and the methodological quality and certainty of the available evidence.

**Methods:** A search was conducted across Scopus, PsycINFO, MEDLINE, and Web of Science Core Collection databases on 29 October 2025. Studies published in English within the past 20 years were eligible if they examined a drama-based intervention in a trauma-exposed population and reported trauma-related psychological outcomes. Methodological quality was appraised using the Mixed Methods Appraisal Tool and certainty of evidence was assessed using GRADE.

**Results:** Twenty-six studies met inclusion criteria out of 1,461 screened papers. Interventions were grouped as psychodrama, dramatherapy, applied theatre, other drama-based psychotherapeutic interventions, and other. The most commonly examined outcomes were depression, anxiety, and PTSD symptoms. Across intervention types, some studies reported improvements in trauma-related and broader psychological outcomes. However, findings were mixed and frequently constrained by small samples, uncontrolled designs, heterogeneous outcome measurement, limited intervention reporting and inconsistent use of terminology. Certainty of evidence was rated as very low.

**Conclusions:** Drama-based interventions show preliminary potential for supporting trauma-exposed populations, but the evidence base remains limited by substantial heterogeneity and low methodological quality. More rigorous study designs, clearer reporting of intervention content and delivery, and greater consistency in outcome assessment are needed to strengthen the evidence base and clarify how drama-based strategies may contribute to trauma-related mental health care.

## HIGHLIGHTS

- Drama-based interventions are being used with trauma-exposed people across clinical, community, and education settings.
- Across 26 studies, some findings suggested improvements in post-traumatic stress, anxiety, depression, and broader psychological wellbeing.
- The evidence remains uncertain because studies used varied methods, inconsistent terminology, and limited descriptions of the interventions.

## Abstract (es)

**Antecedentes:** Las técnicas basadas en el drama se han implementado cada vez más en poblaciones expuestas al trauma. Sin embargo, la base de evidencia sigue siendo conceptual y metodológicamente heterogénea, con una variación considerable en cómo se define el trauma, cómo se describen las intervenciones y qué resultados relacionados con el trauma se evalúan.

**Objetivo:** Esta revisión tuvo como objetivo sintetizar estudios empíricos que examinan intervenciones basadas en el drama para personas expuestas al trauma. La revisión describe las características de las intervenciones basadas en el drama, los tipos de trauma abordados, el patrón y el rango de los resultados psicológicos relacionados con el trauma evaluados, así como la calidad metodológica y certeza de la evidencia disponible.

**Métodos:** Se realizó una búsqueda en las bases de datos Scopus, PsycINFO, MEDLINE y Web of Science Core Collection el 29 de octubre de 2025. Los estudios publicados en inglés en los últimos 20 años fueron elegibles si examinaban una intervención basada en drama en una población expuesta al trauma y reportaban resultados psicológicos relacionados con el trauma. La calidad metodológica se evaluó utilizando la Herramienta de Evaluación de Métodos Mixtos y la certeza de la evidencia se evaluó usando GRADE.

**Resultados:** Veintiséis estudios cumplieron los criterios de inclusión de un total de 1.461 artículos evaluados. Las intervenciones se agruparon como psicodrama, dramaterapia, teatro aplicado, otras intervenciones psicoterapéuticas basadas en el drama y otras. Los resultados más frecuentemente examinados fueron depresión, ansiedad y síntomas de TEPT. En los distintos tipos de intervención, algunos estudios reportaron mejoras en los resultados psicológicos relacionados con el trauma y en los aspectos psicológicos más amplios. Sin embargo, los hallazgos fueron mixtos y frecuentemente limitados por muestras pequeñas, diseños no controlados, mediciones de resultados heterogéneas, descripción insuficiente de las intervenciones y uso inconsistente de la terminología. La certeza de la evidencia se calificó como muy baja.

**Conclusiones:** Las intervenciones basadas en el drama muestran un potencial preliminar para apoyar a poblaciones expuestas al trauma, pero la base de evidencia sigue limitada por una heterogeneidad sustancial y baja calidad metodológica. Se necesitan diseños de estudio más rigurosos, un reporte más claro del contenido y la ejecución de la intervención, y una mayor coherencia en la evaluación de resultados, para fortalecer la base de evidencia y aclarar cómo las estrategias basadas en el drama pueden contribuir a la atención de la salud mental relacionada con el trauma.

## 1. Introduction

Psychological trauma is a major public health concern with individual, societal, and economic consequences (Bellis et al., 2019; Magruder et al., 2017; Peterson et al., 2023). Trauma and adverse experiences have been associated with poorer overall health (Wegman & Stetler, 2009) and increased risk of a wide range of mental health conditions (McKay et al., 2021). The effects of trauma aggregate into increased demand on service utilisation, increased comorbidity, and intergenerational transmission of risk, contributing to persistent patterns of disadvantage and inequity (Bellis et al., 2019; Fang et al., 2012; Graf et al., 2021; Jonson-Reid et al., 2004). Trauma-related mental health difficulties are also associated with substantial costs, including increased healthcare expenditure (Peterson et al., 2023), reduced workforce participation (Metzler et al., 2017) and productivity losses over time (Merrill et al., 2026). Given the breadth of the individual, societal, and economic consequences, how trauma is defined becomes a central issue in shaping what is studied, measured, and ultimately addressed through intervention.

### 1.1. Conceptualising trauma

Psychological trauma is commonly defined as exposure to events or environments that overwhelm an individual’s capacity to cope and that carry potential for significant psychological harm (Herman, 1992). Diagnostic frameworks such as the DSM-5-TR (American Psychiatric Association, 2022) define traumatic exposure narrowly as events involving actual or threatened death, serious injury, or sexual violence (Criterion A). However, a substantial body of trauma research indicates that this narrow definition of trauma exposure may capture only a subset of experiences associated with psychological harm (Kerig, 2023; NHS Scotland, 2017; Saraiya et al., 2025; Swedo et al., 2024). This matters because a narrow, Criterion A-only definition would exclude many populations and experiences relevant to trauma-related mental health, including adverse childhood experiences and complex or chronic interpersonal trauma, that do not always involve threatened death, serious injury, or sexual violence but are nonetheless associated with significant psychological harm. Accordingly, this review adopts a broader conceptualisation of trauma, encompassing adverse childhood experiences, single-incident trauma, and complex or chronic interpersonal trauma (see Eligibility Criteria, below), in order to capture the diversity of populations and experiences addressed by drama-based interventions in the existing literature.

### 1.2. Drama-based strategies

Drama-based and theatre-based (henceforth referred to as drama-based) strategies have been increasingly implemented in contexts with trauma-exposed populations (Jiang et al., 2023). In simple terms, drama-based strategies use techniques from theatre, such as acting out a memory or an imagined situation in a guided session, to help people process difficult experiences in ways that talking alone may not reach. What distinguishes drama-based approaches from other therapies is their use of enactment, role, embodiment, and shared dramatic action as core therapeutic processes (Berghs et al., 2022; Jiang et al., 2023). Rather than relying primarily on discussion or symbolic representation alone, drama-based approaches invite participants to explore experience through movement, performance, role-taking, improvisation, and interaction with others in enacted or imagined scenes. Such processes are theorised to support mechanisms relevant to trauma recovery, including emotional expression and regulation, meaning-making, integration of experience, interpersonal connection, and somatic embodiment (Berghs et al., 2022; Jiang et al., 2023; Li et al., 2025). These processes encompass known mechanisms of trauma recovery such as expression and regulation of affect (Berghs et al., 2022), narrative integration of events (Crespo & Fernández-Lansac, 2016), and social supports (Calhoun et al., 2022; Keiller et al., 2023).

Guideline-recommended psychotherapies (e.g. trauma-focused cognitive behavioural therapy, prolonged exposure, and eye movement desensitisation and reprocessing) are established and effective treatments for trauma and Post-Traumatic Stress Disorder (PTSD). However, treatment attrition remains a clinically important issue (Cloitre et al., 2004; Schottenbauer et al., 2008), and there is growing interest in interventions that engage bodily, sensory, and relational processes alongside verbal and cognitive work in trauma recovery. Drama-based approaches may represent one such pathway to trauma recovery, given their capacity to engage bodily, sensory, and relational processes directly. Drama-based strategies may support the expression and exploration of traumatic experiences and their psychological sequelae (Kuhfuß et al., 2021; Li et al., 2025; van Marle, 2015) which are not readily addressed through exclusively verbal or cognitively oriented interventions. Importantly, these approaches are often implemented in group-based or community settings, which may further support relational repair and social reconnection following trauma exposure (Calhoun et al., 2022; Sippel et al., 2024).

There are a diverse range of practices encompassed in drama-based interventions for trauma, including professionally accredited therapeutic modalities (dramatherapy, psychodrama) as well as more general educational or community treatment programmes utilising applied theatre. In dramatherapy, therapists guide participants through scripted or improvised scenes that reflect their inner experiences or offer opportunities to rehearse new ways of coping to develop insight (Jones, 2010). Psychodrama, on the other hand, is a structured programme (originating with Jacob Moreno) which involves group members acting out each other’s problems in role-play (Moreno, 1993). In a typical session, the therapist (director) helps a participant dramatise a life event as if it were happening in the present, focusing on both actions and feelings. Other group participants (auxiliary egos) play the roles of significant people or aspects of the protagonist’s inner world, while the remaining group observes and later processes the enactment (López-González et al., 2021; Moreno, 1993). Unlike dramatherapy, psychodrama is often more formally structured with these stages and roles prescribed by Moreno’s methodology.

Other approaches sometimes called ‘trauma-informed theatre’ use elements of theatre without necessarily being led by accredited drama or psychology professionals (Keisari et al., 2020). What unites these diverse methods is the use of creative performance and embodiment with the goal of improving mental health symptoms. These approaches may be facilitated by educators, artists, or community workers without formal clinical or drama therapy training. Evidence for the effectiveness of these broader approaches varies and is evaluated in detail in the Results below. Importantly, the present review does not assume equivalence between clinically delivered psychotherapies and community- or education-based drama programmes. Rather, it examines how different forms of drama-based strategies are used across contexts. Accordingly, this review adopted a broad definition of drama-based strategies to reflect the diversity of intervention forms and delivery contexts evident in the existing literature.

Although meta-analytic syntheses indicate promising mental health effects from drama-based therapies (Orkibi et al., 2025), evidence on drama-based approaches specifically for trauma remains uncertain. Existing systematic reviews typically focus on specific age-groups (Berghs et al., 2022), modalities (Berghs et al., 2022; Orkibi & Feniger-Schaal, 2019) or general creative arts (Baker et al., 2018; Morison et al., 2022) and to date, no systematic review has synthesised the evidence on a wide range of drama-based interventions in trauma-exposed populations. Moreover, the field remains limited by a lack of clarity regarding the specific practices and processes that constitute drama-based trauma interventions. The present systematic review was undertaken to address these gaps by synthesising the empirical literature on drama-based interventions for individuals exposed to trauma or adverse experiences.

### 1.3. The aim of this review

The objective of this systematic review was to synthesise empirical studies examining drama-based interventions for individuals exposed to trauma. Specifically, the review aimed to describe the characteristics of drama-based interventions, the types of trauma and adversity addressed, and the pattern and range of trauma-related psychological outcomes assessed. The review also aimed to assess the quality of evidence in the field and highlight areas for future research.

## 2. Methods

### 2.1. Search strategy

This review was preregistered in line with PRISMA guidelines (Prospero ID: CRD420251165033). A comprehensive systematic search strategy was developed to identify studies examining the use of drama-based strategies in relation to psychological trauma. Electronic searches were conducted across four databases: Scopus, PsycINFO, MEDLINE, and Web of Science Core Collection. All database searches were completed on 29 October 2025.

Search terms combined concepts related to drama-based practices (e.g. drama, theatre, role-play, performance-based interventions) with concepts related to trauma, adverse experiences, and trauma-related psychological outcomes. Controlled vocabulary and free-text terms were used. Searches were limited to studies published in English within the past 20 years. The full search strategies for each database are presented in Supplementary tables 1 and 2. Backward citation searching was conducted on included studies.

### 2.2. Eligibility criteria

Studies were eligible for inclusion if they (a) examined drama-based interventions in populations exposed to (b) psychological trauma inclusive of adverse childhood experiences (ACEs), single-incident traumas, or complex traumas and (c) reported at least one psychological or psychosocial outcome relevant to trauma or adversity, regardless of whether the outcome measure itself was trauma-specific. Eligible interventions included drama-based, theatre-based practices including dramatherapy, psychodrama and applied theatre approaches. Studies that did not involve a drama-based component were excluded.

Outcomes of interest included those that reflected psychological processes or symptoms plausibly related to trauma or adversity regardless of whether PTSD was explicitly assessed or diagnosed. We did not require outcome measures to be trauma-specific instruments. Instead, an outcome counted as trauma-related when it was assessed in a study population that met the exposure or diagnosis criteria above. Three considerations supported this approach. First, trauma exposure without a formal PTSD diagnosis still frequently produces clinically significant symptoms. Subthreshold PTSD research found elevated rates of comorbid depression and anxiety, functional impairment, and healthcare use among people who did not meet full diagnostic criteria (McLaughlin et al., 2015). Second, psychological trauma acts as a transdiagnostic risk factor for mood and anxiety presentations and not only for PTSD. A recent umbrella meta-analysis found trauma exposure roughly tripled the odds of any mental disorder, and comorbidity was the norm rather than the exception in trauma-exposed samples (Hogg et al., 2023). Third, complex trauma often presents through what Herman (1992) termed disguised presentations such as anxiety, depression, or somatic symptoms without a discrete PTSD picture. This pattern is consistent with several included studies that captured trauma-related distress through general psychological measures rather than a confirmed PTSD diagnosis (e.g. Campbell Kirk, 2015). This approach also matches practice elsewhere in the drama-based intervention literature. Quinlan et al. (2016), included in this review, deliberately assessed broader emotional and behavioural constructs rather than PTSD symptomatology alone, drawing on the broader creative-arts-therapy literature documenting effects across depression, anxiety, peer relationships, and general wellbeing.

Trauma was conceptualised as encompassing adverse experiences across the lifespan, including ACEs, single-incident trauma, and complex or chronic interpersonal trauma. This framing is largely consistent with the conceptualisation from the NHS Scotland (2017) that highlights that many adversities, particularly experiences of emotional, physical, or sexual abuse may simultaneously constitute both ACEs and traumatic exposures, reflecting the overlapping and cumulative nature of adversity across development. Accordingly, populations meeting criterion (b) via documented displacement or refugee status were considered to satisfy this criterion without requiring a further individually reported exposure type. Refugee status is a legally defined classification requiring documented evidence of a well-founded fear of persecution (UNHCR, 1951), and refugee and forcibly displaced populations show substantially elevated rates of PTSD, depression, and anxiety relative to the general population, consistent with population-level trauma exposure (Fazel et al., 2005; Steel et al., 2009). Studies that did not assess trauma or trauma-related mental health outcomes were excluded.

Studies which reported observational or anecdotal outcomes by authors were also excluded. Only peer-reviewed empirical studies and dissertations were eligible for inclusion. Reviews, opinion pieces, conference abstracts, unpublished studies, and other non-empirical outputs were excluded. No exclusions were applied based on comparator conditions, age ranges, settings, or study design. Studies were restricted to English and published in the last 20 years. This time frame was selected to capture contemporary literature and to align with the review’s PROSPERO-registered search date restriction (from 1 January 2005) and reflects the period during which drama-based and creative arts-based approaches to trauma have been most actively developed, implemented, and evaluated in the peer-reviewed literature.

### 2.3. Data screening and extraction

Title and abstract screening was conducted by one reviewer (EO). A subset of records was independently double screened by a second reviewer to assess consistency. Full-text screening was conducted independently by two reviewers with overlapping allocation (EO 100%, LB 70%, LG 23%, RE 7%). Discrepancies at both stages were resolved through discussion.

Extracted data included study characteristics (authors, year, country), participant characteristics (sample size, age, gender, trauma exposure) intervention characteristics (type of drama-based approach, duration, frequency), comparator characteristics (where applicable), study design, outcome measures, time points, and key findings. Data extraction was conducted by one reviewer (EO) in Covidence, and an additional reviewer (LB) independently cross checked all data extraction against the original articles using Excel. Discrepancies were resolved through discussion. For classification purposes, interventions were coded as psychodrama, dramatherapy, applied theatre, other psychotherapeutic drama-based, or other.

### 2.4. Methodological quality assessment

Methodological quality was assessed using the Mixed Methods Appraisal Tool (MMAT), Version 2018 (Hong et al., 2018). The MMAT was selected because the included studies comprised qualitative, quantitative, mixed methods, and case-based designs. Appraisal criteria were operationalised in a structured Excel spreadsheet. Quality assessment was undertaken by two reviewers (EO & LG), who completed the assessment independently. Any discrepancies were resolved through discussion. Average interrater agreement was 90.3%, details are provided in Supplementary Table 3. The full results of the MMAT appraisal for each included study are presented in Supplementary Figures 1–5 and Supplementary Table 4.

#### 2.4.1. Reporting bias and certainty of evidence

The certainty of the body of evidence was evaluated using the Grading of Recommendations Assessment, Development and Evaluation (GRADE) approach. GRADE assessments were conducted in a structured Excel spreadsheet and assessed risk of bias, inconsistency, indirectness, imprecision, and potential publication bias. GRADE certainty ratings were restricted to studies reporting a comparative effect estimate (randomised and non-randomised controlled designs); single-arm studies without a comparator (case series, case reports, and pre–post designs) do not permit a comparative effect estimate and were therefore synthesised narratively rather than assigned a GRADE certainty rating. The assessment was completed by one reviewer (EO). Although results are synthesised below by intervention type, outcome-level certainty assessments were undertaken among the comparative studies, for the three most commonly reported domains: PTSD, anxiety, and depression (see Supplementary Table 5). These outcomes were selected because they were represented by a sufficient number of studies to permit meaningful cross-study synthesis.

#### 2.4.2. Synthesis

Due to substantial heterogeneity across the included studies, meta-analysis was not undertaken. Instead, findings were synthesised narratively, following the Synthesis Without Meta-analysis (SWiM) reporting guideline (Campbell et al., 2020), consistent with the review’s registered protocol. Studies were grouped by the primary intervention modality described by study authors. However, the terminology and naming conventions used to describe drama-based interventions were inconsistent across studies, and programme descriptions often lacked clarity regarding what specific practices were implemented. Accordingly, these categories were treated as pragmatic groupings for the purposes of narrative synthesis rather than as mutually exclusive or conceptually discrete intervention types. This approach allowed the review to examine how different programme models were implemented in practice, while recognising overlap in terminology and content.

Within each intervention category, studies were compared descriptively in relation to programme characteristics, target populations, trauma context, outcomes assessed, and the pattern of reported findings. Both quantitative and qualitative findings were considered in the synthesis. The review team did not conduct independent thematic or content analysis of primary study data. Instead, quantitative findings were extracted and summarised according to each study’s own reported results and direction of change, and qualitative findings were extracted and summarised according to the themes, perceived effects, and participant-reported experiences already reported by the original study authors.

## 3. Results

### 3.1. Study selection

The PRISMA flow diagram is presented in Figure 1. Initial database searches yielded 2,916 records. Following duplicate removal in EndNote (*n* = 1,335), 1,581 records were imported into Covidence. A further 142 duplicates were identified and removed in Covidence (22 manually; 120 automatically), resulting in 1,439 records for title and abstract screening.

**Figure 1.**
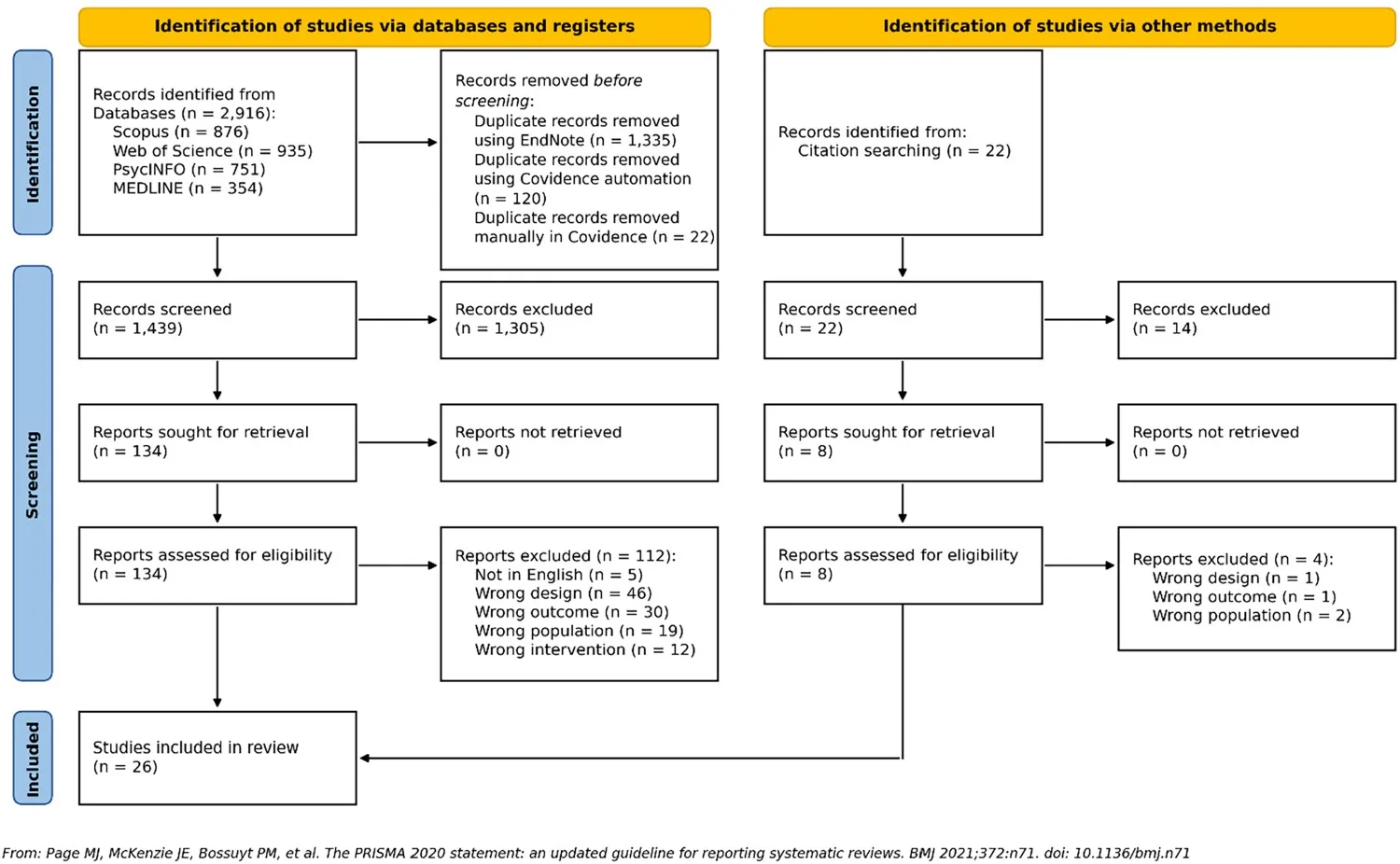
PRISMA 2020 flow diagram of study identification, screening and inclusion.

Title and abstract screening were conducted for 1,439 records, resulting in the exclusion of 1,305 studies that did not meet eligibility criteria. Full-text reports were sought for 134 studies, all of which were successfully retrieved. During full-text screening, 112 studies were excluded. Reasons for exclusion included publication in a language other than English (*n* = 5), absence of a drama-based intervention (*n* = 12), absence of a trauma population (*n* = 19), absence of trauma-related outcomes (*n* = 30), and ineligible study design or output type (*n* = 46). A total of 22 studies met the inclusion criteria and were included in the final review.

An additional 22 studies were identified through backwards citation searching of the 22 included studies. These 22 records were also screened for titles and abstracts in Covidence, 8 studies were identified for full text review, and 4 met inclusion criteria. This brought the total number of studies included in this review to 26.

Screening was conducted in full by one reviewer (EO). A subset of records (*n* = 643; 40.7%) were double screened at title and abstract stage to assess inter-rater reliability. Agreement between reviewers was high (overall proportionate agreement = 85.7%), with a weighted Cohen’s kappa of 0.44, indicating moderate reliability.

### 3.2. Characteristics of studies and of drama programmes

Twenty-six studies were included in this review. Study characteristics are presented in Table 1. Using MMAT design categories, the included studies comprised quantitative non-randomised studies (*n* = 9, 35%), randomised controlled trials (*n* = 3, 12%), mixed methods studies (*n* = 8, 31%), qualitative studies (*n* = 4, 15%), and quantitative descriptive studies (*n* = 2, 8%). Ten studies (38%) included a comparator or control group. Of these, six used an active comparator and six included a passive comparator (e.g. treatment as usual, waitlist, or non-participating control), noting that some studies included more than one comparator type. Studies were conducted across 14 countries, with one study spanning two countries. The largest number were conducted in the United States (*n* = 10, 38%), followed by Lebanon (*n* = 3, 12%), Nigeria (*n* = 2, 8%), and Italy (*n* = 2, 8%), with one study each conducted in the United Kingdom, Austria, Germany and Bulgaria, Türkiye, Spain, Korea, Australia, the Netherlands, and China.

**Table 1.** Characteristics of included studies.

Study	Country	Intervention	Delivery	Number of sessions	Session length (minutes)	Duration of programme	Follow up time points	Setting	Facilitator
Ali and Wolfert (2016)	United States	Other psychotherapeutic	Group	10	120	10	none	Community	Other: military veterans and experts from the VCPA with experience in participating in and delivering the DE-CRUIT treatment.
Allen and Solomon (2012)	United States	Applied Theatre	Group; Face to face	1	90	Single session	Short term (<6 months)	Community	Multidisciplinary
Anichebe et al. (2024)	Nigeria	Dramatherapy	Group; Face to face	12	55–60	6	none	School / Education	Drama Therapist / Art Therapist
Asher et al. (2021)	United States	Other: Improvisation	Group; Face to face	6	90	6	Short term (<6 months)	NR	Theatre Practitioner / Artist; Multidisciplinary
Campbell Kirk (2015)	UK	Dramatherapy	One on one / Individual; Face to face	Up to 12	50	Up to 12	none	Community	Drama Therapist / Art Therapist
Canazei and Fürst (2025)	Austria	Psychodrama	One on one / Individual; Face to face	54	50 min	2 years approximately	Long Term (>12 months)	Clinical / Healthcare	Drama Therapist / Art Therapist; Researcher
Esparza (2016)	United States	Other psychotherapeutic (expressive arts)	Group; Face to face	8	90	8	none	Community	Researcher
Gatta et al. (2010)	Italy	Psychodrama	Group; Face to face	12	75	12 weeks	none	Clinical / Healthcare	Psychologist; Drama Therapist / Art Therapist
Gever et al. (2023)	Nigeria	Dramatherapy	Group; Online	7	90	NR	none	Online / Remote	Drama Therapist / Art Therapist
Giacomucci et al. (2022)	United States	Psychodrama	Group; Face to face	1 × 3 d session	1 d	3 days	Short term (<6 months)	NR	Other: Trained facilitator
Gotseva-Balgaranova et al. (2020)	Germany and Bulgaria	Other psychotherapeutic	Group; Face to face	9	NR	3–8 weeks	none	NR	Other: Trained professionals (can be social workers, psychotherapists)
Karabilgin et al. (2012)	Türkiye	Psychodrama	Group; Face to face	10	6 h	NR (2008–2009)	none	NR	Multidisciplinary - Psychiatrists who were also Drama Therapist / Art Therapist
Kreidie et al. (2024)	Lebanon	Dramatherapy	Group; Face to face	12	60–90	6–12 weeks	none	Community	Drama Therapist / Art Therapist
Little (2017)	United States	Psychodrama; included yoga and mindfulness	Group; Face to face	10	120 min	10	none	School / Education	Multidisciplinary: researcher, counsellor, yoga instructor
McAdam and Johnson (2018)	United States	Dramatherapy	One on one / Individual; Face to face	6–8	15–20 min	8 weeks (two months)	none	School / Education	Drama Therapist / Art Therapist
Miguel and Pino-Juste (2021)	Spain	Other psychotherapeutic	Group; Face to face	20	120	October to July	none	Community	Multidisciplinary: clinical psychologist-researcher
Munjuluri et al. (2020)	United States	Applied Theatre: Playback Theatre	Group; Face to face	4	120 mins	6 including questionnaires and MRIs 4 weeks of PT	none	Community	Not reported
Park and Kim (2022)	Korea	Psychodrama based on Dayton and Van Der Kolk	One on one / Individual; Face to face	5	120 min	5	none	NR	Researcher
Perry et al. (2016)	United States	Psychodrama (Therapeutic spiral)	Group; Face to face	1 workshop	1 d	3 days	Short term (<6 months)	Community	Multidisciplinary: Researcher and TSM certified team
Quinlan et al. (2016)	Australia	Other: Multi Arts Including Drama	Group; Face to face	10	60	10	none	School / Education	Theatre Practitioner / Artist: Psychotherapists
Sakhi et al. (2020)	Lebanon	Dramatherapy	Group; Face to face	12	180	3 months	none	Community	Drama Therapist / Art Therapist
Sakhi et al. (2022)	Lebanon	Dramatherapy	Group; Face to face	12	180	12	none	Community	Drama Therapist / Art Therapist
Sullivan (2021)	United States	Other psychotherapeutic	Group; Face to face	22	NR	school year	None	Community	Multidisciplinary
Testoni et al. (2018)	Italy	Psychodrama	Group; Face to face	weekly sessions for six months	120	6 months	Long Term (>12 months)	Community	Multidisciplinary; Psychotherapist and two support psychodrama trainees
van den Broek (2024)	Netherlands	Dramatherapy with Schema Therapy	One on one / Individual; Face to face	75	NR	Several years with periods of interruption	none	Forensic	Drama Therapist / Art Therapist
Yu et al. (2022)	China	Psychodrama	Group; Face to face	3 sessions x 4 days	NR	6 months	none	NR	NR

Note: NR: not reported; PT: Playback Theatre; MRI: Magnetic Resonance Imaging.

A range of drama-based interventions were identified, including psychodrama (*n* = 9, 35%), dramatherapy (*n* = 8, 31%), applied theatre (*n* = 2, 8%), other psychotherapeutic drama-based approaches (*n* = 5, 19%), and other approaches such as improvisation or multi-arts programmes that included a drama component (*n* = 2, 8%). Programme duration ranged from a single session to delivery over several years, and session lengths ranged from 15 min to full-day formats. Most studies were conducted in community settings (*n* = 10, 38%), followed by school or education settings (*n* = 3, 12%) and clinical or healthcare settings (*n* = 2, 8%). One study took place online and one in a forensic setting, while seven studies did not clearly report the setting. Five studies reported short-term follow-up and two reported longer-term follow-up; the remaining 19 assessed outcomes at post-intervention only. A wide range of outcome measures was used across studies, which are summarised in Supplementary Table 6.

### 3.3. Sample characteristics

Sample characteristics of the included studies are presented in Table 2. Across the included studies, *n* = 1,234 individuals were reported to have been exposed to trauma and/or adverse experiences. In studies that included mixed groups (trauma-exposed and non-trauma-exposed), only data for trauma-exposed participants were extracted where possible. Participants had a range of psychiatric diagnoses, including PTSD, borderline personality disorder, depression/major depressive disorder, substance use disorder, and other psychiatric presentations; however, most studies (*n* = 16) did not report a formal diagnostic category for participants. Study populations were diverse and included community samples (*n* = 10, 38%), clinical samples (*n* = 7, 27%), combined veteran and clinical samples (*n* = 2, 8%), veterans (*n* = 1, 4%), students (*n* = 2, 8%), refugees (*n* = 2, 8%), evacuees (*n* = 1, 4%), and forensic populations (*n* = 1, 4%). Reported trauma exposures also varied widely and included adverse experiences, single-incident traumas, and more complex or chronic traumas.

**Table 2.** Sample characteristics of included studies.

Study	Number of participants with trauma exposure (total *N*)	Number of participants in drama-based programme	Psychiatric Diagnosis	Population descriptor	Adverse Experiences and Traumas	Age Mean (SD) intervention; comparator where reported	Age Range	% Female Intervention
Ali and Wolfert (2016)	3	3	NR	Veterans	Military trauma	NR	NR	33%
Allen and Solomon (2012)	60	20	NR	Community	Chronic Exposure to community violence	12.4 (NR)	9–15	58%
Anichebe et al. (2024)	348	120	NR	Community	Natural Disaster	NR	10–16	NR
Asher et al. (2021)	17	17	NR	Clinical	Life threatening illness / injury	50.8 (12.9)	NR	100%
Campbell Kirk (2015)	15	11	NR	Community	Domestic violence / Intimate partner violence; Witnessing parental violence	Intervention: 28.0 (NR); Control 37.0 (NR)	NR	100%
Canazei and Fürst (2025)	1	1	BPD	Clinical	Neglect; Parental separation; Parental illness	NR	NR	0%
Esparza (2016)	4	4	PTSD	Veterans; Clinical	Military trauma	NR	NR	25%
Gatta et al. (2010)	3 (6)	3	Anxiety; Depression/MDD; ‘immature personality disorder, phobic syndrome, psychotic syndrome, obsessive-compulsive syndrome’	Clinical	Parental Separation; Parental Illness	17	16–18	33%
Gever et al. (2023)	330	55	NR	Evacuees	War as a civilian	NR	25–32 ‘predominately’	Intervention: 33%; Control: 39%
Giacomucci et al. (2022)	17	6 or 7 (inconsistently reported)	PTSD	Clinical	Neglect; Childhood Sexual Abuse; Childhood Emotional Abuse; Childhood Physical Abuse; Sexual Abuse (adulthood)	40.8 (NR)	27–56	0%
Gotseva-Balgaranova et al. (2020)	31	31	NR	Community	War as a civilian; Refugees	Children: 7.9 (1.4); Parents: NR	Children: 6–11; Parents: NR	‘Children: 53%; Parents: 100%’
Karabilgin et al. (2012)	7	7	NR	Clinical	Life threatening illness / injury	33.2 (7.4)	NR	29%
Kreidie et al. (2024)	138	138	NR	Community	War as a civilian; Refugees	NR	18–55	100%
Little (2017)	8	8	NR	Students	Neglect; Childhood Sexual Abuse; Childhood Emotional Abuse; Childhood Physical Abuse; Sexual Abuse (adulthood); Domestic Violence / Intimate Partner Violence	NR	18–32	88%
McAdam and Johnson (2018)	2	2	PTSD, MDD	Community	Childhood Sexual Abuse; Childhood Emotional Abuse; Childhood Physical Abuse; witnessing parental violence; Other: Unstable childhood living (removed by family services)	15.5	15–16	50%
Miguel and Pino-Juste (2021)	17	17	NR	Community	Domestic Violence / Intimate Partner Violence	49 (NR)	21–52	100%
Munjuluri et al. (2020)	13	13	NR	Community	Natural Disaster	48.2 (NR)	NR	38%
Park and Kim (2022)	1	1	NR	Community	Childhood Physical Abuse; Family Violence; Other: Living out of home – facility for abuse victims as a child	15 (NA)	NR	100%
Perry et al. (2016)	13	7	PTSD	Veterans; Clinical	Sexual Abuse (adulthood); military trauma	NR	30–60	100%
Quinlan et al. (2016)	42	22	NR	Students	Other: Refugees	Intervention: 15.9 (1.3); Control: 15.0 (1.8)	NR	60%
Sakhi et al. (2020)	7	7	NR	Refugees	War as a civilian	NR	16–45	100%
Sakhi et al. (2022)	59	59	NR	Refugees	War as a civilian	39 (NR)	18–71	100%
Sullivan (2021)	47	27	PTSD; Anxiety; Depression/MDD; RAD; dissociative disorders; BP; psychotic disorders; autism; ADHD	Clinical	Neglect; Impaired caregiver; Childhood Sexual Abuse; Childhood Emotional Abuse; Childhood Physical Abuse; Sexual Abuse (adulthood); Domestic Violence / Intimate Partner Violence	Intervention: 17.6 (NR); Control: 18.2 (NR)	14–22	62%
Testoni et al. (2018)	4	4	SUD	Clinical	Neglect; Parental Substance Misuse; Parental Illness; Other: Living out of home due to parental inability (boarding school); Childhood Sexual Abuse; Childhood Physical Abuse; Other: Nonspecified child abuse, incarceration, homelessness; Other: Forced abortion	41.25 (NR)	32–50	100%
van den Broek (2024)	1	1	BPD	Forensic	Parental Substance Misuse	54 (NA)	54	0%
Yu et al. (2022)	46	29	Depression/MDD	Community	Not Specified	Intervention: 26.0 (7.2); Control: 28.2 (6.2)	18–50	Intervention:76%; Comparator: 88%

Note: Borderline Personality Disorder (BPD), Post-Traumatic Stress Disorder (PTSD), Major Depressive Disorder (MDD), Reactive Attachment Disorder (RAD), Bipolar Disorder (BP), Attention Deficit Hyperactivity Disorder (ADHD), Substance Use Disorder (SUD), Number or participants were extracted for the drama interventions only where possible and excluded attrition. For Little (2017), 8 participants began the intervention but only 5 completed the programme and provided post-group data; the N reported reflects participants who began the intervention

### 3.4. Trauma-related outcomes studied

Table 3 presents all psychological and psychosocial outcomes assessed across the included studies. For the purposes of the narrative synthesis, we focused on trauma and related mental health outcomes, such as PTSD symptoms, anxiety, depression, psychological distress, dissociation, and closely related emotional or behavioural difficulties. Process, acceptability, and therapy-experience outcomes were reported in some studies, although not synthesised as a focus of this review. The most commonly assessed trauma-related domains were PTSD symptoms, anxiety, and depression. These outcomes were assessed using a range of validated self-report and clinician-administered measures, and in some studies were also explored qualitatively.

**Table 3.** All outcomes assessed in the included studies.

Study	Quantitative outcomes assessed	Qualitative outcomes/themes explored
Ali and Wolfert (2016)	–	Posttraumatic stress experiences; self-awareness; positive psychological change
Allen and Solomon (2012)	Anxiety; stress; coping strategies; violence-avoidance self-efficacy	–
Anichebe et al. (2024)	PTSD symptoms; anxiety	–
Asher et al. (2021)	Quality of life/well-being; loneliness; anxiety; depression; fatigue; social participation/roles; physical function; pain interference; sleep disturbance	–
Campbell Kirk (2015)	Psychological distress and functioning	Goal attainment; client feedback on therapy experience
Canazei and Fürst (2025)	Psychological distress; well-being; symptoms; interpersonal relationships; incongruence; depressive symptoms; personality disorder/personality style scores; emotional regulation; work-related stress; substance use (PQ items)	therapeutic alliance/change process themes
Esparza (2016)	PTSD symptoms; trauma-related guilt	Creative coping; self-reflection; emotional regulation; self-understanding; openness-to-experience; avoidance; trust/support/camaraderie
Gatta et al. (2010)	Anxiety; depression; anger/hostility; overall psychiatric symptoms/mental health	–
Gever et al. (2023)	PTSD symptoms	–
Giacomucci et al. (2022)	PTSD symptoms; spontaneity; post-traumatic growth	–
Gotseva-Balgaranova et al. (2020)	PTSD symptoms; depression; dissociation; anxiety	–
Karabilgin et al. (2012)	Quality of life/mental health	relationships; insight/self-awareness; coping skills
Kreidie et al. (2024)	PTSD symptoms; anxiety; depression	–
Little (2017)	–	Connection with self and other survivors; interpersonal development; intrapersonal development; learning skills/techniques; coping with PTSD symptoms; moving forward
McAdam and Johnson (2018)	Depressive symptoms, including author-derived PTSD-related and depression-only symptom subsets	Teacher and administrator reports of classroom behaviour; therapist observations
Miguel and Pino-Juste (2021)	PTSD symptoms; depression; anxiety; self-esteem; quality of life; life purpose; sexist attitudes/stereotypes	Personal and social competencies; elaboration of trauma
Munjuluri et al. (2020)	PTSD symptoms; anxiety; depression	–
Park and Kim (2022)	PTSD symptoms; anxiety	Awareness of emotions and body reactions; integration of cognition, emotion, and bodily sensation; physical symptoms; self-control/emotional expression
Perry et al. (2016)	Hopelessness; self-esteem	Occupational participation/roles; outlook on the future; relationships; insight into traumatic experiences; tools to support role participation
Quinlan et al. (2016)	Anxiety/depression-related emotional distress; emotional symptoms; behavioural difficulties	–
Sakhi et al. (2020)	–	Negative affect; catharsis; anger regulation; outlook/finding happiness; emotional release through physical activity; self-confidence and openness; friendship/shared experience; sense of community
Sakhi et al. (2022)	–	Emotional regulation; sense of belonging; benefits of movement and exercise; changes within the family
Sullivan (2021)	Depression; dissociation/complex trauma-related symptoms; aggression/social problems; physiological regulation/flexibility	Movement from absence to presence; safety/stabilisation; identification; play; self-disclosure; problem-solving/meaning-making; identity shift; advocacy
Testoni et al. (2018)	Psychological distress/well-being; spontaneity; self-efficacy; hope/optimism/pessimism; personality factors	Helpful and hindering aspects of therapy; change processes and empowerment, including self-awareness and relationships
van den Broek (2024)	Mental health symptoms/functioning; recidivism risk; early maladaptive schemas; schema modes/emotional states	Aggression/conflict-seeking; relationships; contact with vulnerability; loneliness; depressive symptoms; self-esteem still developing
Yu et al. (2022)	Depression; anxiety; coping style	–

Note: Quantitative outcomes were coded from the measures reported in each study and are presented here as broad outcome domains rather than as individual instruments. Qualitative outcomes/themes were included only where studies used qualitative methods or explicitly reported qualitative findings. For Gotseva-Balgaranova et al. (2020), child and parent outcomes were collapsed into a single study row. Post-traumatic growth and trauma-related guilt were treated as distinct constructs rather than as PTSD symptom outcomes. McAdam and Johnson (2018) used author-derived PTSD-related and depression-only symptom subsets rather than a dedicated PTSD instrument.

### 3.5. Critical appraisal of studies

Critical appraisal findings using the MMAT (Hong et al., 2018) are presented in Supplementary Figures 1–5 and Supplementary Table 4. Interrater agreement for MMAT ratings was very high overall, with 15 items meeting 100% agreement, overall agreement ranged from 62.5% to 100% (mean: 90.3%), pooled Cohen’s Kappa = 0.84, with variability in agreement relating to confounding, intervention fidelity, and adherence to the quality criteria of each component tradition.

Overall methodological quality varied across study designs. Among the four qualitative studies, methodological quality ranged from strong to mixed. While most qualitative studies demonstrated clear research questions and interpretations that were sufficiently substantiated by the data, adequacy of qualitative data collection methods was rated as unclear (‘can’t tell’) for two studies (Ali & Wolfert, 2016; Sakhi et al., 2020), and adequacy of derivation of findings from the data was rated as unclear for two studies (Sakhi et al., 2020; Sakhi et al., 2022), largely reflecting limited reporting of data collection or analytic procedures in the original publications. Coherence between data sources, analysis, and interpretation was also inconsistent, with one study (Ali & Wolfert, 2016) not meeting this criterion overall. The three randomised controlled trials all met the MMAT screening criteria (adequate research questions and collected data), but methodological limitations were common across the design-specific criteria. In particular, randomisation procedures and baseline comparability were often unclear, and completeness of outcome data, blinding of outcome assessors, and adherence to the assigned intervention were limited or unclear across studies.

Among the quantitative non-randomised studies, outcome and intervention measures were generally appropriate, but there were frequent concerns about representativeness, incomplete or unclear outcome data, and, most notably, lack of control for confounding variables. The two quantitative descriptive studies showed adequate measurement and low apparent risk of nonresponse bias, but were limited by concerns regarding sampling, representativeness, and the appropriateness of statistical analysis. The mixed methods studies generally demonstrated an adequate rationale for using mixed methods and effective integration of qualitative and quantitative components, and most adequately addressed the outputs of that integration. However, there was limited evidence that the different components consistently met the quality criteria of their respective methodological traditions, and justification for divergences or inconsistencies between components was often unclear. Taken together, these findings suggest that although the evidence base shows methodological strengths in some domains, widespread limitations in design quality and reporting reduce confidence in the robustness and causal interpretability of the overall findings.

### 3.6. Certainty of evidence

Detailed GRADE ratings and justifications for each outcome are presented in Supplementary Table 5. GRADE certainty was assessed using only the subset of studies reporting a comparative effect estimate for each outcome (PTSD: *k* = 3, *N* = 695; anxiety: *k* = 5, *N* = 508; depression: *k* = 2, *N* = 88); single-arm studies were synthesised narratively (see Synthesis, above) and excluded from this rating, as were qualitative findings. Certainty of evidence was rated as very low for all three outcomes, reflecting serious risk of bias (confounding was not accounted for in any non-randomised comparative study), serious inconsistency, and very serious imprecision (small samples, no reported confidence intervals). Indirectness was assessed as not serious; publication bias was not formally assessed. The pattern of findings also differed by outcome: for PTSD, all three comparative studies reported symptom reduction, though designs varied greatly in rigour. For anxiety, four of the five comparative studies reported a null, borderline, or non-significant between-group result. Anichebe et al. (2024) reported a significant multivariate effect but this reflected the combined PTSD and GAD outcome set across all three groups rather than an anxiety-specific, drama-specific effect; drama and music therapy did not differ significantly from each other on anxiety symptoms. For depression, neither of the two comparative studies demonstrated a statistically robust between-group benefit at a conventional significance threshold.

### 3.7. Synthesis of individual studies by intervention type

#### 3.7.1. Dramatherapy

Eight studies (31%) (Anichebe et al., 2024; Campbell Kirk, 2015; Gever et al., 2023; Kreidie et al., 2024; McAdam & Johnson, 2018; Sakhi et al., 2020, 2022; van den Broek, 2024) examined dramatherapy interventions. Across these studies, dramatherapy referred to programmes, typically facilitated by trained dramatherapists, in which participants worked through scripted or improvised dramatic scenes to reflect on their experiences and rehearse new ways of coping, consistent with the definition outlined in the Introduction (Jones, 2010). The extent to which programmes adhered to formal dramatherapy training and methodology varied across studies, and in one case dramatherapy was delivered in combination with another therapeutic approach, schema therapy (van den Broek, 2024).

Most (*n* =   5) were group-based and delivered face-to-face in community or education contexts, including school-based delivery for flood-affected children (Anichebe et al., 2024) and community programmes for refugee women (Kreidie et al., 2024; Sakhi et al., 2020, 2022). Two studies (Sakhi et al., 2020, 2022) described dramatherapy in refugee camp contexts in Lebanon, using a consistent programme structure and qualitative evaluation. One study (Gever et al., 2023) utilised a social media–based (online) group dramatherapy programme for evacuees affected by war. Two studies used individual formats. They included a brief individual dramatherapy model for domestic abuse survivors compared with a key worker intervention (Campbell Kirk, 2015), and a forensic case illustration integrating dramatherapy with schema therapy over an extended period (van den Broek, 2024). McAdam and Johnson (2018) reported a small case series examining a trauma-centred dramatherapy approach with adolescents diagnosed with PTSD.

Trauma-related outcomes assessed included PTSD symptoms, anxiety, depression, psychological distress, and broader indicators of emotional and interpersonal functioning. Across the three dramatherapy studies with quantitative outcomes (varying in design rigour: one RCT, one quasi-experimental, and one single-arm pre–post study), PTSD symptoms consistently decreased, while effects on anxiety and depression were more variable. Kreidie et al. (2024), an uncontrolled pre–post study, reported reductions in PTSD and depression, but not anxiety, among socioeconomically disadvantaged and refugee women. In the randomised controlled trial by Anichebe et al. (2024), dramatherapy was associated with reductions in PTSD and anxiety symptoms among flood-affected children relative to comparison conditions. Gever et al. (2023) reported reductions in PTSD symptoms for the dramatherapy arm relative to comparator conditions. Both of these studies had methodological shortcomings, so results should be interpreted cautiously.

Campbell Kirk (2015) compared individual dramatherapy with individual key-worker sessions and found dramatherapy produced significantly greater reduction in general psychological distress scores than the key-worker control condition, though the control group also improved. McAdam and Johnson (2018) described reductions in depressive symptoms in two adolescents following trauma-centred dramatherapy, noting this study consisted of a very small case series. Qualitative studies conducted by Sakhi et al. (2020, 2022) reported perceived benefits among refugee women and girls, including reduced negative affect, and improved emotional regulation, among participants in dramatherapy programmes. In the case study by van den Broek (2024), dramatherapy integrated with schema therapy produced mixed outcomes: aggression and interpersonal aggression decreased, but loneliness increased and mild depressive symptoms persisted.

#### 3.7.2. Psychodrama

Nine studies (35%) (Canazei & Fürst, 2025; Gatta et al., 2010; Giacomucci et al., 2022; Karabilgin et al., 2012; Little, 2017; Park & Kim, 2022; Perry et al., 2016; Testoni et al., 2018; Yu et al., 2022) examined psychodrama interventions. Across these studies, psychodrama referred to therapeutic programmes that used action-based and experiential methods to explore traumatic or psychologically distressing experiences. The interventions typically involved enactment, role work, emotional expression, interpersonal exploration, and embodied processing, although the extent to which they adhered to classical psychodrama traditions varied. In several cases, programmes incorporated additional therapeutic components or were described using specific adaptations such as Therapeutic Spiral Model psychodrama (Giacomucci et al., 2022; Perry et al., 2016), analytical psychodrama (Gatta et al., 2010), or psychodrama delivered alongside adjunctive approaches such as mindfulness, yoga, or pharmacological treatment (Little, 2017; Yu et al., 2022).

Most psychodrama interventions were group-based and delivered face-to-face (*n* = 7, 78%). Settings, populations, and dosage varied considerably. Group interventions were conducted in clinical, community, educational, and therapeutic community settings. The populations included adolescents with psychiatric disorders in hospital services (Gatta et al., 2010), men with trauma histories (Giacomucci et al., 2022), women service members with PTSD (Perry et al., 2016), people living with HIV/AIDS (Karabilgin et al., 2012), adults in substance use treatment communities with documented histories of childhood abuse, neglect, and parental alcoholism (Testoni et al., 2018), and adults with childhood trauma-associated major depressive disorder receiving antidepressants (Yu et al., 2022). Little (2017) examined a university-based trauma group that used psychodrama as the core modality alongside yoga and mindfulness. Two studies used individual formats, including a single-case psychodrama intervention for an abused adolescent (Park & Kim, 2022) and a long-term hermeneutic single-case study for a patient with borderline personality disorder and trauma-related developmental adversity (Canazei & Fürst, 2025). Intervention dose ranged from very brief workshop models delivered across one to three days (Giacomucci et al., 2022; Perry et al., 2016; Yu et al., 2022) to weekly psychodrama over several months (Testoni et al., 2018) or long-term individual therapy over approximately two years (Canazei & Fürst, 2025).

Trauma-related outcomes assessed across studies included PTSD symptoms, anxiety, depression, borderline personality features, post-traumatic growth, and broader psychological distress. Quantitative findings were generally suggestive of benefit, but were heterogeneous and strongly constrained by study design. Among the more structured comparative studies, the most consistent evidence of benefit was for PTSD symptom reduction (Giacomucci et al., 2022). For anxiety and depression specifically, comparative results were mixed to null (Gatta et al., 2010; Yu et al., 2022) and did not consistently indicate an intervention-specific effect. Giacomucci et al. (2022) reported reductions in PTSD symptoms and increases in post-traumatic growth following a Therapeutic Spiral Model workshop relative to a non-participating control group. However, this study reported no inferential statistical tests and used a non-randomised, self-selected comparison group, limiting the strength of this evidence. Yu et al. (2022) found that both the psychodrama-plus-antidepressants and health-education-plus-antidepressants groups improved significantly over time on anxiety and depression measures; however, the post-hoc between-group comparison found no significant difference in depression or anxiety scores at endpoint, with only coping style differing significantly between groups. Separately, concurrent antidepressant use in both arms limits attribution of change to the psychodrama component specifically. Gatta et al. (2010) reported significant reductions in several psychiatric symptom domains at the group level, but the individual trajectories of the three trauma-exposed adolescents were mixed.

Smaller uncontrolled and single-case studies also described improvements in trauma-related and related mental health outcomes, including psychological distress, depression, anxiety, trauma symptoms, and emotional regulation (Canazei & Fürst, 2025; Karabilgin et al., 2012; Park & Kim, 2022; Testoni et al., 2018). Qualitative findings from Little (2017) suggested broader participant-reported changes, including trauma-related meaning-making. Perry et al. (2016) reported improvements in related mental health outcomes including hopelessness and self-esteem among female service members participating in a Therapeutic Spiral Model workshop, although PTSD-specific outcomes were not clearly reported as a primary quantified endpoint. Karabilgin et al. (2012) reported qualitative reductions in depressive feelings alongside a significant improvement on the SF-36 mental health subscale among people living with HIV/AIDS.

#### 3.7.3. Applied theatre

Two studies were synthesised under applied theatre: a single-session educational-entertainment theatre intervention for Black adolescents exposed to community violence (Allen & Solomon, 2012), and a four-session playback theatre intervention for adults affected by Hurricane Harvey (Munjuluri et al., 2020). Playback theatre was included within this category because it is understood as a form of applied theatre (Wright, 2013). Although the two studies differed substantially in population, trauma context, and intervention intensity, both used theatre as a participatory, socially situated process rather than as an individualised clinical treatment.

The impact of the interventions on trauma-related outcomes was mixed. In Allen and Solomon (2012), the intervention was associated with improved violence-avoidance self-efficacy and anxiety symptom reductions pre- to post-test; however, comparisons to a control group were not significant. In the study by Munjuluri et al. (2020), playback theatre was associated with significant pre–post reductions in anxiety and PTSD symptoms, whereas depressive symptoms did not significantly improve.

#### 3.7.4. Other drama-based psychotherapeutic interventions

Five studies (19%) were synthesised as other drama-based psychotherapeutic interventions (Ali & Wolfert, 2016; Esparza, 2016; Gotseva-Balgaranova et al., 2020; Miguel & Pino-Juste, 2021; Sullivan, 2021). Across these studies, theatrical or drama-informed processes were embedded within programmes that were framed as psychotherapeutic or psychosocial interventions, although none were labelled as psychodrama or dramatherapy studies. Instead, the interventions combined elements of theatre practice with established therapeutic approaches such as cognitive processing therapy, narrative therapy, person-centred expressive arts therapy, trauma stabilisation programmes, and trauma-focused models.

In the study by Ali and Wolfert (2016), the DE-CRUIT programme utilised a psychotherapy model for military veterans that integrated classical actor training, Shakespearean text work, and trauma-focused therapeutic techniques. Similarly working with veteran populations, Esparza (2016) described a person-centred expressive arts group therapy programme incorporating dramatic and creative arts processes within a group psychotherapy framework for combat-related PTSD. Two studies (Gotseva-Balgaranova et al., 2020; Miguel & Pino-Juste, 2021) focused on civilian populations exposed to interpersonal or collective trauma. Miguel and Pino-Juste (2021) evaluated a group programme for women who had experienced intimate partner violence that combined elements of dramatherapy, psychodrama, and Theatre of the Oppressed. Gotseva-Balgaranova et al. (2020) evaluated the Evidence-Based Trauma Stabilisation programme delivered to refugee families which incorporated structured group activities and expressive processes, including drama-based techniques. Sullivan (2021) described a Trauma Drama intervention implemented within a residential treatment setting for youth with extensive trauma histories and complex mental health presentations. The programme involved a phased treatment where participants were encouraged to rehearse alternative responses to traumatic situations and explore personal experiences within a structured dramatic framework.

Despite differences in population, setting, and theoretical orientation, several common features were evident across the interventions. All programmes were delivered in group formats and relied on structured dramatic or expressive activities, such as improvisation, role play, narrative construction, and symbolic enactment, to facilitate engagement with traumatic experiences. In most cases, these activities were embedded within broader psychotherapeutic frameworks that emphasised safety, emotional expression, and interpersonal connection rather than direct exposure to traumatic memories alone. The use of fictional scenarios, metaphor, or aesthetic distance was frequently described as allowing participants to explore traumatic material in a contained and manageable manner.

Across the five studies, quantitative outcomes primarily assessed symptoms of PTSD and depression. The pattern of findings was mixed and the strength of evidence varied substantially across studies. In Sullivan (2021), the study reported significant pre–post decreases in PTSD and depression scores within the treatment group (with a *p* < .10 threshold given the small sample), while between-group comparisons remained inconclusive. Improvements in depression were also observed in the study by Miguel and Pino-Juste (2021), although changes in PTSD symptoms were not significant.

Evidence from the remaining studies was more tentative and largely descriptive. Ali and Wolfert (2016) presented a small case series of three veterans; each veteran showed a reduction in individual PTSD Checklist (PCL) scores meeting a validated ‘positive response to treatment’ threshold, though no group-level statistical analysis was conducted. Esparza (2016) reported individual pre–post reductions in clinician-rated PTSD symptoms and trauma-related guilt, together with qualitative improvements in anger and emotional regulation but again did not report group-level quantitative analyses. Findings from Gotseva-Balgaranova et al. (2020) were mixed; among children, there was a significant reduction in child self-reported PTSD intrusions and in several parent-reported child symptom domains, including depression, PTSD arousal, and dissociation, while other child symptom domains showed non-significant decreases.

#### 3.7.5. Other

Two studies (8%) in this review were categorised as other interventions. They comprised a six-week improvisational theatre programme for breast cancer survivors (Asher et al., 2021) and a school-based multi-arts creative therapy programme for adolescents from refugee backgrounds (Quinlan et al., 2016). These interventions were grouped separately because neither fit appropriately within the other categories. Asher et al. (2021) evaluated a structured improv programme centred on spontaneity, trust, and group interaction, while Quinlan et al. (2016) examined a broader creative arts therapy model integrating art, music, storytelling, and drama within a school mental health service.

The two studies differed substantially in modality, population, and therapeutic structure. In one study (Asher et al., 2021), participants attended six 90-minute improv classes focused on trust and listening, group mind, character, and scene work. In contrast, the other study (Quinlan et al., 2016) utilised a HEAL programme, a multimodal creative arts therapy intervention delivered by qualified therapists and incorporating sculpture, painting, drawing, music, storytelling, and drama, alongside narrative and strengths-based approaches. Students accessed at least one session per week over one school term, with some also receiving individual therapy.

Findings across the two studies were mixed. In Asher et al. (2021), the improv intervention was associated with significant improvements in anxiety and depression that were maintained at one-month follow-up, however this was a small uncontrolled study. In the study by Quinlan et al. (2016), self-reported anxiety, and depression did not significantly differ between groups.

## 4. Discussion

This review synthesised 26 studies examining drama-based interventions delivered to trauma-exposed populations across diverse settings and participant groups. Although some studies reported improvements in trauma-related psychological outcomes, the evidence base was heterogeneous, methodologically limited, and of very low certainty. Findings were frequently constrained by methodological limitations, including small samples, uncontrolled or non-randomised designs, case-based evaluations, and inconsistent outcome measurement. Substantial variation was also observed in intervention structure and duration. Taken together, this heterogeneity limits direct comparison across studies and precludes firm conclusions regarding effectiveness. These findings are broadly consistent with previous systematic reviews and meta-analyses of drama- and arts-based interventions for mental health (Baker et al., 2018; Berghs et al., 2022; Morison et al., 2022; Orkibi et al., 2025; Orkibi & Feniger-Schaal, 2019), which similarly report heterogeneous effects and methodological limitations, although the present review highlights that these challenges persist within trauma-exposed populations specifically.

A notable feature of the literature was the diversity of interventions represented. These approaches appeared to differ in their theoretical framing, delivery context, and degree of formal therapeutic structure. In broad terms, psychodrama studies tended to emphasise structured enactment, role work, and action-based exploration within group psychotherapy contexts, whereas dramatherapy studies more often described improvisation, storytelling, and symbolic enactment within wider expressive or psychotherapeutic frameworks. Applied theatre and improvisation-based programmes were more often implemented in educational or community settings and tended to emphasise participatory storytelling, collective reflection, or social engagement rather than formal clinical treatment. These distinctions likely reflect the multidisciplinary origins of drama-based work, but they also contribute to fragmented terminology and unclear boundaries between approaches, making comparison across studies difficult.

Despite these differences in terminology and theoretical orientation, many of the interventions reviewed appeared to draw on recurring dramatic practices such as enactment of personal experiences, role exploration, storytelling, improvisation, and embodied expression through movement or performance. Group interaction and shared witnessing were also commonly described, with participants observing or responding to enacted scenes, narratives, or dramatic exercises within a group setting. Although these practices were embedded within different intervention traditions, their recurrence across study descriptions suggests that drama-based trauma interventions may involve overlapping procedural elements even when they are described using different labels.

These recurring practices may be relevant because they provide experiential ways of engaging with narratives, emotions, and interpersonal dynamics relevant to trauma recovery. Role enactment and storytelling may allow difficult experiences to be represented and reflected on in structured or symbolic forms, consistent with theoretical accounts of narrative integration (Crespo & Fernández-Lansac, 2016) and with cognitive models that treat narrative reorganisation of trauma memory as central to post-traumatic recovery (Brewin et al., 1996; Ehlers & Clark, 2000). The embodied and movement-based elements of these practices may engage bodily and affect-regulation processes emphasised in somatic approaches to trauma (Berghs et al., 2022; Kuhfuß et al., 2021), consistent with evidence linking interoceptive awareness to improved emotion regulation and reduced hyperarousal (Leech et al., 2024; Ogden et al., 2006). Group witnessing may create opportunities for interpersonal recognition and shared reflection, consistent with the role of social support in trauma recovery (Calhoun et al., 2022; Keiller et al., 2023) and with theoretical accounts of trauma recovery as fundamentally relational (Herman, 1992). However, intervention descriptions were too limited to identify mechanisms of change or determine which practices were responsible for observed outcomes, and no included study tested a mechanism directly. At most, the activities described across programmes overlap conceptually with therapeutic processes recognised in trauma treatment, including narrative exploration, emotional expression, and relational engagement within a supportive group context. These connections remain largely implicit rather than directly examined within the literature, though mechanism-focused work has begun elsewhere in the creative arts therapies more broadly (de Witte et al., 2021) and has not yet been extended specifically to trauma-exposed populations.

These findings also carry potential clinical and broader relevance. Guideline-recommended trauma therapies such as TF-CBT, PE, and EMDR are effective for many people, but treatment attrition remains a persistent clinical problem (Cloitre et al., 2004; Schottenbauer et al., 2008), and trauma-related mental health difficulties continue to impose substantial individual, service, and economic burden (Bellis et al., 2019; Peterson et al., 2023). Drama-based approaches, by engaging bodily, expressive, and relational processes alongside or instead of verbal disclosure, may offer an alternative or adjunctive pathway for individuals who disengage from, or do not respond to, standard talk-based therapies, and may be particularly suited to group, community, and educational settings where formal clinical services are limited or difficult to access. At the same time, the very low certainty of the current evidence base means these potential benefits remain provisional, and should not yet inform treatment recommendations or service commissioning decisions without further rigorous evaluation.

The ability to interpret these patterns is limited by several methodological and reporting constraints within the existing evidence base. Many studies relied on small samples and uncontrolled or non-randomised designs, and only a minority included comparator groups. Follow-up periods were inconsistent and often limited to immediate post-intervention assessment. Outcome measurement also varied substantially across studies, with a wide range of instruments used to assess trauma-related outcomes. In addition to these methodological limitations, descriptions of the interventions themselves were frequently brief or inconsistent. Many studies provided limited information regarding session activities, facilitator training, or programme structure, and relied on readers' assumed knowledge of interventions making it difficult to determine what specific practices occurred within sessions or how interventions differed in practice. This lack of detailed reporting restricts interpretation, limits replicability, and makes it difficult to identify which aspects of programmes may be most relevant to observed outcomes.

This review should also be interpreted in light of several limitations in the review process itself. First, studies were restricted to those published in English within the past 20 years, which may have resulted in the exclusion of relevant studies published in other languages or outside this time frame. Second, although multiple major databases were searched and backward citation searching was undertaken, additional sources such as trial registers, grey literature databases, organisational websites, journal hand-searching, and forward citation searching were not used. As a result, some relevant studies may not have been captured. Additionally, the substantial heterogeneity across study designs, interventions, populations, and outcomes precluded meta-analysis and limited the extent to which findings could be directly compared across studies. Accordingly, the conclusions of this review should be interpreted as a narrative synthesis of a diverse and methodologically heterogeneous literature, rather than a definitive estimate of intervention effectiveness.

These findings highlight several priorities for future research. Greater transparency in reporting intervention content, facilitator training, delivery format, and adherence procedures would improve interpretability and allow more meaningful comparison across programmes/studies. More rigorous evaluation designs, including controlled trials, more consistent outcome measurement, and longer follow-up periods, are also needed to better assess the effects and durability of these approaches. Future research may also benefit from examining more systematically how specific dramatic practices are used within trauma interventions and mapping dramatic practice to other more widely used therapy modalities for trauma treatment. Although programmes are often described using distinct intervention labels, the recurrence of practices such as enactment, storytelling, role exploration, and embodied group activities across study descriptions suggests that these may represent shared features of drama-based trauma work. Clarifying how such practices are implemented, described, and evaluated may contribute to a more precise conceptualisation of drama-based approaches within trauma-related mental health research and show the unique contribution these approaches can offer to complement other therapies.

## 5. Conclusion

To our knowledge, this systematic review was the first to synthesise the existing evidence base for drama-based interventions in improving mental health symptoms in trauma-exposed populations. It identified a diverse and methodologically heterogeneous body of literature. Across psychodrama, dramatherapy, applied theatre, and other drama-based approaches, some studies reported improvements in trauma-related psychological outcomes, including post-traumatic stress symptoms, anxiety, and depression. However, the evidence base was characterised by substantial variation in intervention type, trauma context, outcome measurement, terminology, and study design, and certainty of evidence for the most commonly examined outcomes was very low.

The findings of this review suggest that drama-based approaches may hold promise as flexible and potentially valuable non-cognitive-based therapy interventions for trauma-exposed populations across clinical, educational, and community settings, but current evidence does not permit strong conclusions regarding effectiveness or comparative benefit. Future research would benefit from more rigorous study designs, greater consistency in outcome assessment, longer follow-up, and clearer reporting of intervention content, facilitation, and implementation. Improved specification of the dramatic strategies used within programmes may also help clarify how these interventions are conceptualised and how they may contribute to trauma-related mental health care.

## Generative artificial intelligence

Microsoft Copilot was used to support language editing and phrasing suggestions during manuscript preparation.

## Supplementary Material

1007 Supplementary materials_TRACKED.docx

0205 Supplementary materials.docx

## Data Availability

Data sharing is not applicable to this article because this study is a systematic review of published literature. Data extracted and analysed for the review are available in the supplementary materials.

## References

[CIT0001] Ali, A., & Wolfert, S. (2016). Theatre as a treatment for posttraumatic stress in military veterans: Exploring the psychotherapeutic potential of mimetic induction. *The Arts in Psychotherapy*, *50*, 58–65. 10.1016/j.aip.2016.06.004

[CIT0002] Allen, V. D., & Solomon, P. (2012). Educational-entertainment as an intervention with black adolescents exposed to community violence. *Journal of Prevention & Intervention in the Community*, *40*(4), 313–324. 10.1080/10852352.2012.707452 22970784

[CIT0003] American Psychiatric Association. (2022). *Diagnostic and statistical manual of mental disorders* (5 Text Revision ed.). 10.1176/appi.books.9780890425787.

[CIT0004] Anichebe, O., Anibueze, A. U., Anum, V., Ohaja, E. U., Ezeugwu, C. A., Obasi, N. T., Onogwu, E. O., & Gever, V. C. (2024). Effectiveness of drama and music therapies as health communication interventions for reducing anxiety and posttraumatic disorders among children-victims of flood. *Complementary Therapies in Clinical Practice*, *57*, 101890. 10.1016/j.ctcp.2024.101890 39106584

[CIT0005] Asher, A., Kim, S., Diniz, M. A., Mack, A., & Shirazipour, C. H. (2021). The impact of improvisational theater/comedy on well-being among breast cancer survivors: A pilot feasibility study. *Alternative and Complementary Therapies*, *27*(6), 276–284. 10.1089/act.2021.29360.aas

[CIT0006] Baker, F. A., Metcalf, O., Varker, T., & O'Donnell, M. (2018). A systematic review of the efficacy of creative arts therapies in the treatment of adults with PTSD. *Psychological Trauma*, *10*(6), 643–651. 10.1037/tra0000353 29199839

[CIT0007] Bellis, M. A., Hughes, K., Ford, K., Ramos Rodriguez, G., Sethi, D., & Passmore, J. (2019). Life course health consequences and associated annual costs of adverse childhood experiences across Europe and North America: A systematic review and meta-analysis. *The Lancet Public Health*, *4*(10), e517–e528. 10.1016/s2468-2667(19)30145-8 PMC7098477 31492648

[CIT0008] Berghs, M., Prick, A., Vissers, C., & van Hooren, S. (2022). Drama therapy for children and adolescents with psychosocial problems: A systemic review on effects, means, therapeutic attitude, and supposed mechanisms of change. *Children*, *9*(9), Article 1358. 10.3390/children9091358 PMC9497558 36138667

[CIT0009] Brewin, C. R., Dalgleish, T., & Joseph, S. (1996). A dual representation theory of posttraumatic stress disorder. *Psychological Review*, *103*(4), 670–686. 10.1037/0033-295x.103.4.670 8888651

[CIT0010] Calhoun, C. D., Stone, K. J., Cobb, A. R., Patterson, M. W., Danielson, C. K., & Bendezú, J. J. (2022). The role of social support in coping with psychological trauma: An integrated biopsychosocial model for posttraumatic stress recovery. *Psychiatric Quarterly*, *93*(4), 949–970. 10.1007/s11126-022-10003-w PMC9534006 36199000

[CIT0011] Campbell, M., McKenzie, J. E., Sowden, A., Katikireddi, S. V., Brennan, S. E., Ellis, S., Hartmann-Boyce, J., Ryan, R., Shepperd, S., Thomas, J., Welch, V., & Thomson, H. (2020). Synthesis without meta-analysis (SWiM) in systematic reviews: Reporting guideline. *BMJ*, *368*, l6890. 10.1136/bmj.l6890 PMC7190266 31948937

[CIT0012] Campbell Kirk, J. (2015). Dramatherapy with women survivors of domestic abuse: A small scale research study. *Dramatherapy*, *37*(1), 28–43. 10.1080/02630672.2015.1101480

[CIT0013] Canazei, M., & Fürst, J. (2025). Individual psychodrama therapy with an auxiliary ego for borderline personality disorder: A hermeneutic single-case efficacy design study. *Zeitschrift für Psychodrama und Soziometrie*, *24*(2), 251–270. 10.1007/s11620-025-00857-0

[CIT0014] Cloitre, M., Stovall-McClough, K. C., Miranda, R., & Chemtob, C. M. (2004). Therapeutic alliance, negative mood regulation, and treatment outcome in child abuse-related posttraumatic stress disorder. *Journal of Consulting and Clinical Psychology*, *72*(3), 411–416. 10.1037/0022-006x.72.3.411 15279525

[CIT0015] Crespo, M., & Fernández-Lansac, V. (2016). Memory and narrative of traumatic events: A literature review. *Psychological Trauma: Theory, Research, Practice, and Policy*, *8*(2), 149–156. 10.1037/tra0000041 25915647

[CIT0016] de Witte, M., Orkibi, H., Zarate, R., Karkou, V., Sajnani, N., Malhotra, B., Ho, R. T. H., Kaimal, G., Baker, F. A., & Koch, S. C. (2021). From therapeutic factors to mechanisms of change in the creative arts therapies: A scoping review. *Frontiers in Psychology*, *12*, Article 678397. 10.3389/fpsyg.2021.678397 PMC8336579 34366998

[CIT0017] Ehlers, A., & Clark, D. M. (2000). A cognitive model of posttraumatic stress disorder. *Behaviour Research and Therapy*, *38*(4), 319–345. 10.1016/s0005-7967(99)00123-0 10761279

[CIT0018] Esparza, J. (2016). *Using person-centered expressive arts group therapy with combat-related PTSD veterans* [Doctoral dissertation, Saybrook University]. ProQuest Dissertations and Theses Global (Publication No. 3724918). https://www.proquest.com/openview/fd4396e9a42f94e1fd814387a4fa3550/1?pq-origsite=gscholar&cbl=18750&diss=y

[CIT0019] Fang, X., Brown, D. S., Florence, C. S., & Mercy, J. A. (2012). The economic burden of child maltreatment in the United States and implications for prevention. *Child Abuse & Neglect*, *36*(2), 156–165. 10.1016/j.chiabu.2011.10.006 PMC3776454 22300910

[CIT0020] Fazel, M., Wheeler, J., & Danesh, J. (2005). Prevalence of serious mental disorder in 7000 refugees resettled in western countries: A systematic review. *Lancet*, *365*(9467), 1309–1314. 10.1016/S0140-6736(05)61027-6 15823380

[CIT0021] Gatta, M., Dal Zotto, L., Del Col, L., Spoto, A., Testa Costantino, P., Ceranto, G., Sorgato, R., Bonafede, C., & Battistella, P. (2010). Analytical psychodrama with adolescents suffering from psycho-behavioral disorder: Short-term effects on psychiatric symptoms. *The Arts in Psychotherapy*, *37*(3), 240–247. 10.1016/j.aip.2010.04.010

[CIT0022] Gever, V. C., Iyendo, T. O., Obiugo-Muoh, U. O., Okunade, J. K., Agujiobi-Odoh, N., Udengwu, N., Talabi, F. O., & Nwokolo, P. N. (2023). Comparing the effect of social media-based drama, music and art therapies on reduction in post-traumatic symptoms among Nigerian refugees of Russia's invasion of Ukraine. *Journal of Pediatric Nursing*, *68*, e96–e102. 10.1016/j.pedn.2022.11.018 36470757

[CIT0023] Giacomucci, S., Marquit, J., & Walsh, K. M. (2022). A controlled pilot study on the effects of a therapeutic spiral model trauma-focused psychodrama workshop on post-traumatic stress, spontaneity and post-traumatic growth. *Zeitschrift für Psychodrama und Soziometrie*, *21*(1), 171–188. 10.1007/s11620-022-00665-w

[CIT0024] Gotseva-Balgaranova, K., Popivanov, I. D., & Mutafchieva, M. (2020). Impact evaluation of the evidence-based trauma stabilisation programme for refugee, asylum seeking and immigrant families. *Zeitschrift für Psychodrama und Soziometrie*, *19*(Suppl 1), 147–160. 10.1007/s11620-020-00568-8

[CIT0025] Graf, G. H., Chihuri, S., Blow, M., & Li, G. (2021). Adverse childhood experiences and justice system contact: A systematic review. *Pediatrics*, *147*(1), Article e2020021030. 10.1542/peds.2020-021030 PMC7786827 33328338

[CIT0026] Herman, J. L. (1992). *Trauma and recovery*. Basic Books.

[CIT0027] Hogg, B., Gardoki-Souto, I., Valiente-Gómez, A., Rosa, A. R., Fortea, L., Radua, J., Amann, B. L., & Moreno-Alcázar, A. (2023). Psychological trauma as a transdiagnostic risk factor for mental disorder: An umbrella meta-analysis. *European Archives of Psychiatry and Clinical Neuroscience*, *273*(2), 397–410. 10.1007/s00406-022-01495-5 36208317

[CIT0028] Hong, Q. N., Fàbregues, S., Bartlett, G., Boardman, F., Cargo, M., Dagenais, P., & … O’Cathain, A. (2018). The Mixed Methods Appraisal Tool (MMAT) version 2018 for information professionals and researchers. *Education for Information*, *34*(4), 285–291. 10.3233/EFI-180221

[CIT0029] Jiang, L., Alizadeh, F., & Cui, W. (2023). Effectiveness of drama-based intervention in improving mental health and well-being: A systematic review and meta-analysis during the COVID-19 pandemic and post-pandemic period. *Healthcare*, *11*(6), Article 839. 10.3390/healthcare11060839 PMC10048456 36981496

[CIT0030] Jones, P. (2010). *Drama as therapy, Vol 2: Clinical work and research into practice*. Routledge.

[CIT0031] Jonson-Reid, M., Drake, B., Kim, J., Porterfield, S., & Han, L. (2004). A prospective analysis of the relationship between reported child maltreatment and special education eligibility among poor children. *Child Maltreatment*, *9*(4), 382–394. 10.1177/1077559504269192 15538037

[CIT0032] Karabilgin, O. S., Gokengin, G., Doganer, I., & Gokengin, D. (2012). The effect of psychodrama on people living with HIV/AIDS. *European Journal of Psychotherapy and Counselling*, *14*(4), 317–333. 10.1080/13642537.2012.734529

[CIT0033] Keiller, E., Tjasink, M., Bourne, J., Ougrin, D., Carr, C. E., & Lau, J. Y. (2023). A systematic review of dramatherapy interventions used to alleviate emotional distress and support the well-being of children and young people aged 8–18 years old. *JCPP Advances*, *3*(3), Article e12145. 10.1002/jcv2.12145 PMC10501692 37720583

[CIT0034] Keisari, S., Gesser-Edelsburg, A., Yaniv, D., & Palgi, Y. (2020). Playback theatre in adult day centers: A creative group intervention for community-dwelling older adults. *PLoS One*, *15*(10), Article e0239812. 10.1371/journal.pone.0239812 PMC7529427 33002024

[CIT0035] Kerig, P. K. (2023). Introduction to the special section: Developmental perspectives on trauma exposure and posttraumatic stress. *Journal of Child & Adolescent Trauma*, *16*(2), 381–390. 10.1007/s40653-023-00557-7 PMC10205950 37234830

[CIT0036] Kreidie, L. H., Sakhi, S., Wardani, F., AlSabah, I., & Anbar, K. (2024). The impact of dramatherapy on the mental health of socioeconomically disadvantaged and refugee women in Lebanon. *Journal of Social Service Research*, *50*(6), 991–1007. 10.1080/01488376.2024.2349236

[CIT0037] Kuhfuß, M., Maldei, T., Hetmanek, A., & Baumann, N. (2021). Somatic experiencing – effectiveness and key factors of a body-oriented trauma therapy: A scoping literature review. *European Journal of Psychotraumatology*, *12*(1), 1929023. 10.1080/20008198.2021.1929023 PMC8276649 34290845

[CIT1001] Leech, K., Stapleton, P., & Patching, A. (2024). A roadmap to understanding interoceptive awareness and post-traumatic stress disorder: A scoping review. *Frontiers in Psychiatry*, *15*, Article 1355442. 10.3389/fpsyt.2024.1355442 PMC11150711 38840943

[CIT0038] Li, Z., Cui, Q., & Liu, X. (2025). Systematic review of the effectiveness of arts therapy for children and adolescents with post-traumatic stress disorder. *Frontiers in Psychiatry*, *16*, Article 1716481. 10.3389/fpsyt.2025.1716481 PMC12739758 41458022

[CIT0039] Little, M. (2017). *Reconnection group for individuals who have experienced interpersonal trauma: An explanatory case study* [Doctoral dissertation, University of Arkansas]. ScholarWorks@UARK. https://scholarworks.uark.edu/etd/1935

[CIT0040] López-González, M. A., Morales-Landazábal, P., & Topa, G. (2021). Psychodrama group therapy for social issues: A systematic review of controlled clinical trials. *International Journal of Environmental Research and Public Health*, *18*(9), Article 4442. 10.3390/ijerph18094442 PMC8122755 33922138

[CIT0041] Magruder, K. M., McLaughlin, K. A., & Elmore Borbon, D. L. (2017). Trauma is a public health issue. *European Journal of Psychotraumatology*, *8*(1), 1375338. 10.1080/20008198.2017.1375338 PMC5800738 29435198

[CIT0042] McAdam, E., & Johnson, D. (2018). Reducing depressive symptoms in adolescents with posttraumatic stress disorder using drama therapy. In A. Zubala, & V. Karkou (Eds.), *Arts therapies in the treatment of depression* (pp. 48–67). Routledge. 10.4324/9781315454412-4.

[CIT0043] McKay, M. T., Cannon, M., Chambers, D., Conroy, R. M., Coughlan, H., Dodd, P., Healy, C., O’Donnell, L., & Clarke, M. C. (2021). Childhood trauma and adult mental disorder: A systematic review and meta-analysis of longitudinal cohort studies. *Acta Psychiatrica Scandinavica*, *143*(3), 189–205. 10.1111/acps.13268 33315268

[CIT0044] McLaughlin, K. A., Peverill, M., Gold, A. L., Alves, S., & Sheridan, M. A. (2015). Child maltreatment and neural systems underlying emotion regulation. *Journal of the American Academy of Child and Adolescent Psychiatry*, *54*(9), 753–762. 10.1016/j.jaac.2015.06.010 PMC4548288 26299297

[CIT0045] Merrill, R. M., Scott, E. N., & Sumsion, L. (2026). Adverse childhood experiences impact unemployment and inability to work among adults in the United States. *Frontiers in Psychology*, *17*, Article 1630105. 10.3389/fpsyg.2026.1630105 PMC12979093 41835879

[CIT0046] Metzler, M., Merrick, M. T., Klevens, J., Ports, K. A., & Ford, D. C. (2017). Adverse childhood experiences and life opportunities: Shifting the narrative. *Children and Youth Services Review*, *72*, 141–149. 10.1016/j.childyouth.2016.10.021 PMC10642285 37961044

[CIT0047] Miguel, M. M. L., & Pino-Juste, M. (2021). Therapeutic achievements of a program based on drama therapy, the theater of the oppressed, and psychodrama with women victims of intimate partner violence. *Violence Against Women*, *27*(9), 1273–1296. 10.1177/1077801220920381 32573368

[CIT0048] Moreno, J. L. (1993). *Psicodrama*. Editora Cultrix.

[CIT0049] Morison, L., Simonds, L., & Stewart, S. F. (2022). Effectiveness of creative arts-based interventions for treating children and adolescents exposed to traumatic events: A systematic review of the quantitative evidence and meta-analysis. *Arts & Health*, *14*(3), 237–262. 10.1080/17533015.2021.2009529 34969356

[CIT0050] Munjuluri, S., Bolin, P. K., Lin, Y. T. A., Garcia, N. L., Gauna, L., Nguyen, T., & Salas, R. (2020). A pilot study on playback theatre as a therapeutic aid after natural disasters: Brain connectivity mechanisms of effects on anxiety. *Chronic Stress*, *4*, Article 2470547020966561. 10.1177/2470547020966561 PMC7643224 33210057

[CIT0051] NHS Scotland. (2017). *Transforming psychological trauma: A knowledge and skills framework for the Scottish Workforce*. https://traumatransformation.scot/app/uploads/2023/09/nationaltraumatrainingframework-final.pdf

[CIT0052] Ogden, P., Minton, K., & Pain, C. (2006). *Trauma and the body: A sensorimotor approach to psychotherapy*. W. W. Norton & Company.

[CIT0053] Orkibi, H., & Feniger-Schaal, R. (2019). Integrative systematic review of psychodrama psychotherapy research: Trends and methodological implications. *PLoS One*, *14*(2), e0212575. 10.1371/journal.pone.0212575 PMC6380607 30779787

[CIT0054] Orkibi, H., Keisari, S., Sajnani, N. L., & de Witte, M. (2025). Effectiveness of drama-based therapies on mental health outcomes: A systematic review and meta-analysis of controlled studies. *Psychology of Aesthetics, Creativity, and the Arts*, *19*(4), 878–895. 10.1037/aca0000582

[CIT0055] Park, A., & Kim, E. (2022). The effect of psychodrama on post-traumatic stress symptoms in abused adolescents: A single case study. *Journal for ReAttach Therapy and Developmental Diversities*, *5*(Special Issue 2), 270–283.

[CIT0056] Perry, R., Saby, K., Wenos, J., Hudgins, K., & Baller, S. (2016). Psychodrama intervention for female service members using the therapeutic spiral model. *The Journal of Psychodrama, Sociometry, and Group Psychotherapy*, *64*(1), 11–23. 10.12926/peszyc83

[CIT0057] Peterson, C., Aslam, M. V., Niolon, P. H., Bacon, S., Bellis, M. A., Mercy, J. A., & Florence, C. (2023). Economic burden of health conditions associated with adverse childhood experiences among US adults. *JAMA Network Open*, *6*(12), e2346323. 10.1001/jamanetworkopen.2023.46323 PMC10701608 38055277

[CIT0058] Quinlan, R., Schweitzer, R. D., Khawaja, N., & Griffin, J. (2016). Evaluation of a school-based creative arts therapy programme for adolescents from refugee backgrounds. *The Arts in Psychotherapy*, *47*, 72–78. 10.1016/j.aip.2015.09.006

[CIT0059] Sakhi, S., Kreidie, L. H., Wardani, F., AlSabah, H. S. I., & Anbar, K. (2020). The power of theater expression and communication: A psychological theraputical intervention in a refugee camp: An IPA study into the narratives of women refugees’ experience with drama therapy. *Psychology Research*, *10*(1), 10–23. 10.17265/2159-5542/2020.01.002

[CIT0060] Sakhi, S., Kreidie, L. H., Wardani, F., & Anbar, K. (2022). Drama therapy as a mental health intervention for women in the Shatila refugee camp, Lebanon. *Intervention Journal of Mental Health and Psychosocial Support in Conflict Affected Areas*, *20*(1), 58–64. 10.4103/INTV.INTV_12_20

[CIT0061] Saraiya, T. C., Bauer, A. G., Banks, D. E., Brown, D. G., Jarnecke, A. M., Ebrahimi, C. T., & Bernard, D. L. (2025). Are we gatekeeping trauma? A conceptual model to expand criterion A for invisible, identity-based, and systemic traumas. *Social Science & Medicine*, *375*, 118090. 10.1016/j.socscimed.2025.118090 PMC12700328 40267759

[CIT0062] Schottenbauer, M. A., Glass, C. R., Arnkoff, D. B., Tendick, V., & Gray, S. H. (2008). Nonresponse and dropout rates in outcome studies on PTSD: Review and methodological considerations. *Psychiatry: Interpersonal and Biological Processes*, *71*(2), 134–168. 10.1521/psyc.2008.71.2.134 18573035

[CIT0063] Sippel, L. M., Liebman, R. E., Schäfer, S. K., Ennis, N., Mattern, A. C., Rozek, D. C., & Monson, C. M. (2024). Sources of social support and trauma recovery: Evidence for bidirectional associations from a recently trauma-exposed community sample. *Behavioural Sciences*, *14*(4), Article 284. 10.3390/bs14040284 PMC11047467 38667080

[CIT0064] Steel, Z., Chey, T., Silove, D., Marnane, C., Bryant, R. A., & van Ommeren, M. (2009). Association of torture and other potentially traumatic events with mental health outcomes among populations exposed to mass conflict and displacement: A systematic review and meta-analysis. *JAMA*, *302*(5), 537–549. 10.1001/jama.2009.1132 19654388

[CIT0065] Sullivan, M. (2021). Revising the script: Mixed-method study of trauma drama for complex-trauma-exposed youth in residential treatment [Dissertation, Widener University]. APA PsycInfo. https://www.proquest.com/openview/157bc8d009545f4d46497efa3cb53ba4/1?pq-origsite=gscholar&cbl=18750&diss=y

[CIT0066] Swedo, E., Pampati, S., Anderson, K., Thorne, E., McKinnon, I., Brener, N. D., Stinson, J., Mpofu, J., & Niolon, P. (2024). Adverse childhood experiences and health conditions and risk behaviors among high school students—Youth Risk Behavior Survey, United States, 2023. *MMWR Supplements*, *73*(4), 39–49. 10.15585/mmwr.su7304a5. PMC11559682 39378222

[CIT0067] Testoni, I., Cecchini, C., Zulian, M., Guglielmin, M. S., Ronconi, L., Kirk, K., Berto, F., Guardigli, C., & Cruz, A. S. (2018). Psychodrama in therapeutic communities for drug addiction: A study of four cases investigated using idiographic change process analysis. *The Arts in Psychotherapy*, *61*, 10–20. 10.1016/j.aip.2017.12.007

[CIT0069] UNHCR. (1951). *Convention relating to the status of refugees*. United Nations. https://www.unhcr.org/media/1951-refugee-convention-and-1967-protocol-relating-status-refugees 12289353

[CIT0070] van den Broek, E. (2024). I'm great! I'm no good … .: A case illustration of drama therapeutic work with a male offender of domestic violence in a forensic outpatient setting. *Journal of Clinical Psychology*, *80*(6), 1448–1465. 10.1002/jclp.23674 38470474

[CIT0071] van Marle, H. (2015). PTSD as a memory disorder. *European Journal of Psychotraumatology*, *6*(1), 27633. 10.3402/ejpt.v6.27633

[CIT0072] Wegman, H. L., & Stetler, C. (2009). A meta-analytic review of the effects of childhood abuse on medical outcomes in adulthood. *Biopsychosocial Science and Medicine*, *71*(8), 805–812. 10.1097/PSY.0b013e3181bb2b46 19779142

[CIT0073] Wright, P. (2013). Playback theatre: Learning and enquiry through applied theatre. *Journal of Arts and Communities*, *5*(1), 7–23. 10.1386/jaac.5.1.7_1

[CIT0074] Yu, R.-Q., Tan, H., Wang, E.-D., Huang, J., Wang, P.-J., Li, X.-M., Zheng, H.-H., Lv, F.-J., & Hu, H. (2022). Antidepressants combined with psychodrama improve the coping style and cognitive control network in patients with childhood trauma-associated major depressive disorder. *World Journal of Psychiatry*, *12*(8), 1016–1030. 10.5498/wjp.v12.i8.1016 PMC9476846 36158310

