# Supplementary material for: Investigating the role of drama-based strategies in addressing psychological trauma: a systematic review
Source: Eur J Psychotraumatol. 2026 Oct 20;17(1):2739019. doi: 10.1080/20008066.2026.2739019 (PMC13686220; doi:10.1080/20008066.2026.2739019)
Supplement: Supplementary file 1 [file ZEPT_A_2739019_SM6254.docx]

**Supplementary Table 1**

*Database Search Concepts*

|  | Concept A Trauma and related conditions (combine with OR) | Concept B Drama based strategies  (combine with OR) | Search notes |
| --- | --- | --- | --- |
| MEDLINE Ovid | Use the subject headings for post traumatic stress disorder, acute stress disorder, child abuse, domestic violence, disaster victims, borderline personality disorder, dissociative disorders, depression, anxiety. Include free text terms: post traumatic stress disorder or PTSD, complex post traumatic stress disorder or cPTSD, acute stress disorder, trauma, psychological trauma, childhood trauma, childhood abuse, adverse childhood experiences, interpersonal violence, family violence, natural disaster, war trauma, refugee trauma, borderline personality disorder or BPD, dissociative disorder, depression, anxiety, adjustment disorder. Combine all trauma terms with OR. | Use the subject headings for drama therapy and psychodrama. Include free text terms: drama therapy, dramatherapy, psychodrama, theatre or theater combined with therapy or intervention or program or performance or applied or community, role play, improvisation, forum theatre, playback theatre, theatre of the oppressed, creative arts therapy or creative arts therapies. | Combine all trauma terms with OR.  Combine all drama terms with OR.  Combine Concepts A AND B.  Exclude animal only records.  Apply limits: English language and year 2005 to current. |
| **Web of science core collection** | “post traumatic stress disorder” OR PTSD OR “complex post traumatic stress disorder” OR cPTSD OR “acute stress disorder” OR trauma OR “psychological trauma” OR “childhood trauma” OR “childhood abuse” OR “adverse childhood experiences” OR “interpersonal violence” OR “family violence” OR “natural disaster*” OR “war trauma” OR “refugee trauma” OR “borderline personality disorder” OR BPD OR “dissociative disorder*” OR depression OR anxiety OR “adjustment disorder”. | “drama therapy” OR dramatherapy OR psychodrama OR (theatre NEAR/3 (therap* OR intervention* OR program* OR performance OR applied OR community)) OR (theater NEAR/3 (therap* OR intervention* OR program* OR performance OR applied OR community)) OR “role play” OR improvisation OR “forum theatre” OR “playback theatre” OR “theatre of the oppressed” OR “creative arts therapy” OR “creative arts therapies”. | As above |
| **APA PsychInfo OVID** | As above | As above | As above |
| **SCOPUS** | Search in title abstract keywords for: post traumatic stress disorder OR PTSD OR complex post traumatic stress disorder OR cPTSD OR acute stress disorder OR trauma OR psychological trauma OR childhood trauma OR childhood abuse OR adverse childhood experiences OR interpersonal violence OR family violence OR natural disaster OR war trauma OR refugee trauma OR borderline personality disorder OR BPD OR dissociative disorder OR depression OR anxiety OR adjustment disorder. | Search in title abstract keywords for: drama therapy OR dramatherapy OR psychodrama OR theatre or theater within three words of therapy or intervention or program or performance or applied or community OR role play OR improvisation OR forum theatre OR playback theatre OR theatre of the oppressed OR creative arts therapy OR creative arts therapies. | Combine Concepts A AND B with AND.  Apply limits in the Scopus filters: English and publication year 2005 to present. |

**Supplementary Table 2**

*Database Search Strings*

| Database | Code |
| --- | --- |
| **OVID Medline** | 1. Stress Disorders, Post Traumatic/  2. post traumatic stress disorder.mp. or PTSD.mp.  3. complex post traumatic stress disorder.mp. or cPTSD.mp.  4. Stress Disorders, Traumatic, Acute/ or acute stress disorder.mp.  5. Child Abuse/ or childhood trauma.mp. or childhood abuse.mp. or adverse childhood experiences.mp.  6. Domestic Violence/ or interpersonal violence.mp. or family violence.mp.  7. Disaster Victims/ or natural disaster*.mp. or war trauma.mp. or refugee trauma.mp.  8. Borderline Personality Disorder/ or BPD.mp.  9. Dissociative Disorders/ or dissociative disorder*.mp.  10. Depression/ or depression.mp. or exp Anxiety Disorders/ or anxiety.mp. or adjustment disorder*.mp.  11. trauma.mp. or psychological trauma.mp.  12. 1 or 2 or 3 or 4 or 5 or 6 or 7 or 8 or 9 or 10 or 11  13. Drama Therapy/ or drama therapy.mp. or dramatherapy.mp.  14. Psychodrama/ or psychodrama.mp.  15. ((theatre or theater) adj3 (therap* or intervention* or program* or performance or applied or community)).ti,ab.  16. role play.ti,ab. or improvisation.ti,ab.  17. forum theatre.ti,ab. or playback theatre.ti,ab. or theatre of the oppressed.ti,ab.  18. creative arts therap*.ti,ab.  19. 13 or 14 or 15 or 16 or 17 or 18  20. 12 and 19  21. animals/ not humans/  22. 20 not 21  23. limit 22 to english language  24. limit 23 to yr="2005 -Current" |
| SCOPUS | TITLE-ABS-KEY(  "post traumatic stress disorder" OR PTSD OR  "complex post traumatic stress disorder" OR cPTSD OR  "acute stress disorder" OR  trauma OR "psychological trauma" OR  "childhood trauma" OR "childhood abuse" OR "adverse childhood experiences" OR  "interpersonal violence" OR "family violence" OR  "natural disaster*" OR "war trauma" OR "refugee trauma" OR  "borderline personality disorder" OR BPD OR  "dissociative disorder*" OR depression OR anxiety OR "adjustment disorder"  )  AND  TITLE-ABS-KEY(  "drama therapy" OR dramatherapy OR psychodrama OR  (theatre W/3 (therap* OR intervention* OR program* OR performance OR applied OR community)) OR  (theater W/3 (therap* OR intervention* OR program* OR performance OR applied OR community)) OR  "role play" OR improvisation OR "forum theatre" OR "playback theatre" OR  "theatre of the oppressed" OR "creative arts therapy" OR "creative arts therapies"  )  AND PUBYEAR > 2004  AND (LIMIT-TO(LANGUAGE, "English")) |
| Web of Science Core Collection | TS=("post traumatic stress disorder" OR PTSD OR "complex post traumatic stress disorder" OR cPTSD OR "acute stress disorder" OR trauma OR "psychological trauma" OR "childhood trauma" OR "childhood abuse" OR "adverse childhood experiences" OR "interpersonal violence" OR "family violence" OR "natural disaster*" OR "war trauma" OR "refugee trauma" OR "borderline personality disorder" OR BPD OR "dissociative disorder*" OR depression OR anxiety OR "adjustment disorder")  AND  TS=("drama therapy" OR dramatherapy OR psychodrama OR (theatre NEAR/3 (therap* OR intervention* OR program* OR performance OR applied OR community)) OR (theater NEAR/3 (therap* OR intervention* OR program* OR performance OR applied OR community)) OR "role play" OR improvisation OR "forum theatre" OR "playback theatre" OR "theatre of the oppressed" OR "creative arts therapy" OR "creative arts therapies")  Refined by: LANGUAGES: (ENGLISH)  Timespan: 2005–2025  Indexes: SCI-EXPANDED, SSCI, A&HCI, ESCI |
| OVID Psych Info | Posttraumatic Stress Disorder/  posttraumatic stress disorder.mp. or "post traumatic stress disorder".mp. or PTSD.mp.  complex posttraumatic stress disorder.mp. or cPTSD.mp.  Acute Stress Disorder/ or acute stress disorder.mp.  Child Abuse/ or childhood trauma.mp. or childhood abuse.mp. or adverse childhood experiences.mp.  Domestic Violence/ or interpersonal violence.mp. or family violence.mp.  Disasters/ or disaster victims.mp. or natural disaster*.mp. or war trauma.mp. or refugee trauma.mp.  Borderline Personality Disorder/ or BPD.mp.  Dissociative Disorders/ or dissociative disorder*.mp.  Depressive Disorders/ or depression.mp. or Anxiety Disorders/ or anxiety.mp. or adjustment disorder*.mp.  trauma.mp. or psychological trauma.mp.  1 or 2 or 3 or 4 or 5 or 6 or 7 or 8 or 9 or 10 or 11  Drama Therapy/ or "drama therapy".mp. or dramatherapy.mp.  Psychodrama/ or psychodrama.mp.  ((theatre or theater) adj3 (therap* or intervention* or program* or performance or applied or community)).ti,ab.  role play.ti,ab. or improvisation.ti,ab.  "forum theatre".ti,ab. or "playback theatre".ti,ab. or "theatre of the oppressed".ti,ab.  "creative arts therap*".ti,ab.  13 or 14 or 15 or 16 or 17 or 18  12 and 19  limit 20 to english language  limit 21 to yr="2005 -Current" |

**Supplementary Table 3**

***Interrater reliability for MMAT***

| **MMAT Criterion** | **Study Type** | ***n*** | ***κ*** | **% Agreement** |
| --- | --- | --- | --- | --- |
| S1 | All | 26 | 1.00 | 100.0 |
| S2 | All | 26 | 0.89 | 95.7 |
| QL1 | Qualitative | 12 | 1.00a | 100.0 |
| QL2 | Qualitative | 12 | 1.00 | 100.0 |
| QL3 | Qualitative | 12 | 0.82 | 91.7 |
| QL4 | Qualitative | 12 | 0.63 | 91.7 |
| QL5 | Qualitative | 12 | 0.58 | 75.0 |
| QR1 | Quantitative RCT | 3 | 1.00a | 100.0 |
| QR2 | Quantitative RCT | 3 | 1.00 | 100.0 |
| QR3 | Quantitative RCT | 3 | 1.00 | 100.0 |
| QR4 | Quantitative RCT | 3 | 1.00 | 100.0 |
| QR5 | Quantitative RCT | 3 | 1.00 | 100.0 |
| QN1 | Quantitative non randomised | 16 | 0.76 | 87.5 |
| QN2 | Quantitative non randomised | 16 | 1.00 | 100.0 |
| QN3 | Quantitative non randomised | 16 | 0.88 | 93.8 |
| QN4 | Quantitative non randomised | 16 | 0.00 | 75.0 |
| QN5 | Quantitative non randomised | 16 | 0.42 | 75.0 |
| QD1 | Quantitative descriptive | 2 | 1.00a | 100.0 |
| QD2 | Quantitative descriptive | 2 | 1.00a | 100.0 |
| QD3 | Quantitative descriptive | 2 | 1.00a | 100.0 |
| QD4 | Quantitative descriptive | 2 | 1.00a | 100.0 |
| QD5 | Quantitative descriptive | 2 | 1.00a | 100.0 |
| MM1 | Mixed methods | 8 | 1.00a | 100.0 |
| MM2 | Mixed methods | 8 | 0.00 | 87.5 |
| MM3 | Mixed methods | 8 | 0.00 | 87.5 |
| MM4 | Mixed methods | 8 | 1.00 | 100.0 |
| MM5 | Mixed methods | 8 | 0.25 | 62.5 |
|  |  |  |  |  |
| *Note.* MMAT = Mixed Methods Appraisal Tool. For 15 criteria where both raters showed perfect agreement with no variance in ratings (S1, QL1, QL2, QR1, QR2, QR3, QR4, QR5, QD1, QD2, QD3, QD4, QD5, MM1, MM4), Cohen's κ could not be calculated; 100% agreement is reported for these items. Values for quantitative nonrandomised and qualitative studies are high than in the reported manuscript because mixed methods studies were also evaluated within these domains. | | | | |

**Supplementary Figure 1**

*MMAT Critical Appraisal of Qualitative Studies*

*Note.* Y= yes, N = no, CT = can’t tell.

**Supplementary Figure 2**

*MMAT Critical Appraisal of Quantitative Randomised Controlled Trials*

*Note.* Y= yes, N = no, CT = can’t tell.

**Supplementary Figure 3**

*MMAT Critical Appraisal of Quantitative Nonrandomized Studies*

*Note.* Y= yes, N = no, CT = can’t tell.

**Supplementary Figure 4**

*MMAT Critical Appraisal of Quantitative Descriptive Studies*

*Note.* Y= yes, N = no, CT = can’t tell.

**Supplementary Figure 5**

*MMAT Critical Appraisal of Mixed Methods Studies*

*Note.* Y= yes, N = no, CT = can’t tell.

**Supplementary Table 4**

*MMAT Quality Appraisal of Included Studies (N = 26)*

| **Study** | **Study design** | **S1** | **S2** | **QL1** | **QL2** | **QL3** | **QL4** | **QL5** | **QR1** | **QR2** | **QR3** | **QR4** | **QR5** | **QN1** | **QN2** | **QN3** | **QN4** | **QN5** | **QD1** | **QD2** | **QD3** | **QD4** | **QD5** | **MM1** | **MM2** | **MM3** | **MM4** | **MM5** |
| --- | --- | --- | --- | --- | --- | --- | --- | --- | --- | --- | --- | --- | --- | --- | --- | --- | --- | --- | --- | --- | --- | --- | --- | --- | --- | --- | --- | --- |
| Ali & Wolfert (2016) | Qualitative research | N | CT | CT | CT | Y | Y | N |  |  |  |  |  |  |  |  |  |  |  |  |  |  |  |  |  |  |  |  |
| Allen & Solomon (2012) | Quantitative non-randomized | Y | N |  |  |  |  |  |  |  |  |  |  | CT | Y | Y | N | CT |  |  |  |  |  |  |  |  |  |  |
| Anichebe et al. (2024) | Quantitative randomized controlled trial | Y | Y |  |  |  |  |  | CT | CT | N | N | N |  |  |  |  |  |  |  |  |  |  |  |  |  |  |  |
| Asher et al. (2021) | Quantitative non-randomized | Y | Y |  |  |  |  |  |  |  |  |  |  | CT | Y | N | N | CT |  |  |  |  |  |  |  |  |  |  |
| Campbell Kirk (2015) | Quantitative non-randomized | Y | Y |  |  |  |  |  |  |  |  |  |  | CT | Y | N | N | N |  |  |  |  |  |  |  |  |  |  |
| Canazei & Furst (2025) | Mixed Methods | Y | N | Y | Y | Y | Y | Y |  |  |  |  |  | N | Y | Y | N | CT |  |  |  |  |  | Y | Y | Y | CT | N |
| Esparza (2016) | Mixed Methods | Y | N | Y | Y | Y | Y | Y |  |  |  |  |  | CT | Y | Y | N | CT |  |  |  |  |  | Y | Y | Y | CT | N |
| Gatta et al. (2010) | Quantitative non-randomized | Y | N |  |  |  |  |  |  |  |  |  |  | N | Y | Y | N | CT |  |  |  |  |  |  |  |  |  |  |
| Giacomucci et al. (2022) | Quantitative non-randomized | N | CT |  |  |  |  |  |  |  |  |  |  | N | Y | N | N | CT |  |  |  |  |  |  |  |  |  |  |
| Gotseva-Balgaranova et al. (2020) | Quantitative non-randomized | Y | N |  |  |  |  |  |  |  |  |  |  | N | CT | Y | N | CT |  |  |  |  |  |  |  |  |  |  |
| Gever et al., (2023) | Quantitative randomized controlled trial | Y | Y |  |  |  |  |  | CT | CT | CT | CT | CT |  |  |  |  |  |  |  |  |  |  |  |  |  |  |  |
| Karabilgin et al. (2012) | Mixed Methods | Y | Y | Y | Y | CT | Y | CT |  |  |  |  |  | N | Y | Y | N | Y |  |  |  |  |  | Y | Y | Y | CT | N |
| Kreidie et al. (2024) | Quantitative non-randomized | Y | Y |  |  |  |  |  |  |  |  |  |  | CT | Y | CT | N | CT |  |  |  |  |  |  |  |  |  |  |
| Little (2017) | Qualitative research | Y | Y | Y | Y | Y | Y | Y |  |  |  |  |  |  |  |  |  |  |  |  |  |  |  |  |  |  |  |  |
| McAdam & Johnson (2018) | Quantitative descriptive | Y | Y |  |  |  |  |  |  |  |  |  |  |  |  |  |  |  | N | N | Y | Y | N |  |  |  |  |  |
| Miguel & Pino-Juste (2021) | Mixed Methods | Y | N | Y | Y | Y | Y | Y |  |  |  |  |  | CT | Y | CT | N | N |  |  |  |  |  | Y | Y | Y | CT | N |
| Munjuluri et al. (2020) | Quantitative non-randomized | Y | Y |  |  |  |  |  |  |  |  |  |  | N | Y | Y | N | CT |  |  |  |  |  |  |  |  |  |  |
| Park & Kim (2022) | Mixed Methods | Y | Y | Y | Y | CT | Y | Y |  |  |  |  |  | N | Y | Y | N | Y |  |  |  |  |  | Y | Y | Y | CT | CT |
| Perry et al. (2016) | Mixed Methods | Y | Y | Y | Y | CT | Y | CT |  |  |  |  |  | N | Y | Y | N | CT |  |  |  |  |  | Y | Y | Y | CT | N |
| Quinlan et al. (2016) | Quantitative non-randomized | Y | Y |  |  |  |  |  |  |  |  |  |  | CT | Y | Y | N | CT |  |  |  |  |  |  |  |  |  |  |
| Sakhi et al. (2020) | Qualitative research | Y | Y | Y | CT | CT | Y | Y |  |  |  |  |  |  |  |  |  |  |  |  |  |  |  |  |  |  |  |  |
| Sakhi et al. (2022) | Qualitative research | Y | Y | Y | Y | CT | Y | Y |  |  |  |  |  |  |  |  |  |  |  |  |  |  |  |  |  |  |  |  |
| Sullivan (2021) | Mixed Methods | Y | Y | Y | Y | Y | Y | Y |  |  |  |  |  | CT | Y | CT | N | CT |  |  |  |  |  | Y | Y | Y | Y | N |
| Testoni et al. (2018) | Mixed Methods | Y | Y | Y | Y | Y | CT | CT |  |  |  |  |  | N | N | Y | N | CT |  |  |  |  |  | Y | Y | Y | Y | N |
| Van Den Broek (2024) | Quantitative descriptive | N | CT |  |  |  |  |  |  |  |  |  |  |  |  |  |  |  | N | N | Y | Y | N |  |  |  |  |  |
| Yu et al. (2022) | Quantitative randomized controlled trial | Y | Y |  |  |  |  |  | CT | Y | N | CT | CT |  |  |  |  |  |  |  |  |  |  |  |  |  |  |  |
|  |  |  |  |  |  |  |  |  |  |  |  |  |  |  |  |  |  |  |  |  |  |  |  |  |  |  |  |  |
| *Note*. Y = Yes, N = No, CT = Can't Tell. S1 = Are there clear research questions?, S2 = Do the collected data allow to address the research questions?, QL1 = Is the qualitative approach appropriate to answer the research question?, QL2= Are the qualitative data collection methods adequate to address the research question? QL3 = Are the findings adequately derived from the data?, QL4 = Is the interpretation of results sufficiently substantiated by the data?, QL5 = Is there coherence between qualitative data sources, collection, analysis and interpretation?, QR1 = Is randomisation appropriately performed?, QR2 = Are the groups comparable at baseline?, QR3 = Are there complete outcome data?, QR4 = Are outcomes assessors blinded to the intervention provided? QR5 = Did the participants adhere to the assigned intervention?, QN1 = Are the participants representative of the target population?, QN2 = Are measurements appropriate regarding both the outcome and intervention (or exposure)?, QN3 = Are there complete outcome data?, QN4 = Are the confounders accounted for in the design and analysis?, QN5 = During the study period, is the intervention administered (or exposure occured) as intended?, QD1 = Is the sampling strategy relevant to address the research question?, QD2 = Is the sample representative of the target population?, QD3 = Are the measurements appropriate?, QD4 = Is the risk of nonresponse bias low?, QD5 = Is the statistical analysis appropriate to answer the research question?, MM1 = Is there an adequate rationale for using a mixed methods design to address the research question?, MM2 = Are the different components of the study effectively integrated to answer the research question?, MM3 = Are the outputs of the integration of qualitative and quantitative components adequately addressed?, MM5 = Do the different components of the study adhere to the quality criteria of each tradition of the methods involved? | | | | | | | | | | | | | | | | | | | | | | | | | | | | |

**Supplementary Table 5**

*GRADE Evidence Profile*

| **Outcome** | **Study design** | **Risk of bias** | **Inconsistency** | **Indirectness** | **Imprecision** | **Publication bias** | **Certainty** |
| --- | --- | --- | --- | --- | --- | --- | --- |
| PTSD | Mixed designs | serious | serious | not serious | serious | Not assessed | Very Low    Very Low    Very Low   |
| Anxiety | Mixed designs | serious | serious | not serious | serious | Not assessed |  |
| Depression | Mixed designs | serious | serious | not serious | serious | Not assessed |  |

Post-intervention PTSD outcomes were reported across 13 included studies with a range of study designs. Across these studies, findings were mixed, with some reporting reductions in PTSD symptoms and others reporting mixed or inconclusive results. Overall certainty of evidence for PTSD was rated as very low using GRADE because of serious risk of bias, inconsistency, and imprecision. Indirectness was not judged serious, and reporting bias was not formally assessed.

Eleven studies reported post-intervention anxiety outcomes across a range of study designs. Findings were mixed overall, with some studies reporting improvement and others reporting mixed or non-significant results. Overall certainty of evidence for anxiety was rated as very low using GRADE because of serious risk of bias, inconsistency, and imprecision. Indirectness was not judged serious, and reporting bias was not formally assessed.

Post-intervention depression outcomes were reported across 12 included studies and intervention categories. Findings were mixed overall, with some studies reporting reductions in depressive symptoms and others reporting mixed or non-significant results. Overall certainty of evidence for depression was rated as very low using GRADE because of serious risk of bias, inconsistency, and imprecision. Indirectness was not judged serious, and reporting bias was not formally assessed.

| **Supplementary Table 6**  *List of trauma related measures used by the studies included in this review, organised by outcome* | |
| --- | --- |
| **Outcome** | **Measure** |
| Anxiety | Spence Anxiety Scale for Children |
|  | Generalised Anxiety Disorder-7 |
|  | Behaviour Assessment Scale Children-3 Anxiety |
|  | Patient-Reported Outcomes Measurement Information System-29 (Anxiety sub scale) |
|  | Symptom Checklist - 90 - Revised, Anxiety and Phobia subscales |
|  | Trauma Symptom Checklist for Young Children, anxiety subscale |
|  | Hamilton Anxiety Rating Scale |
|  | "Revised Children's Manifest Anxiety Scale: Second Editon " |
|  | Beck Anxiety Inventory |
|  | The Hopkins Symptom Checklist Anxiety Subscale |
| PSTD | PSTD Subset of Beck Depression Inventory |
|  | The Severity of PTSD Symptoms Scale |
|  | PTSD Checklist for DSM 5 |
|  | Post Traumatic Diagnostic Scale |
|  | Posttraumatic Stress Disorder Reaction Index |
|  | International Trauma Questionnaire |
|  | Trauma Symptom Checklist for Young Children |
|  | Impact of Events Scale - Revised |
|  | Posttraumatic Symptom Scale-Interview |
| Depression | Patient-Reported Outcomes Measurement Information System Depression subscale |
|  | Symptom Checklist - 90 -Depression Subscale |
|  | Beck Depression Inventory |
|  | Qualitative interviews |
|  | Patient Health Questionnaire -9 |
|  | Quick Inventory of Depressive Symptomatology - Self-Report |
|  | Hopkins Symptom Checklist Depression Subscale |
|  | Children’s Depression Inventory 2 |
|  | Hamilton Depression Scale |
| Overall Mental Health | Wellness Inventory, scales 26-182 |
|  | Symptom Checklist - 90 revised total |
|  | Strengths and Difficulties Questionnaire |
|  | Child Behaviour Check List |
|  | Clinical Outcomes in Routine Evaluation - Outcome Measure Depression and Anxiety subscales |
| Other | Violence avoidance - Penn Violence Avoidance Self-Efficacy Scale |
|  | Stress - Multicultural Events Schedule for Adolescents |
|  | Trauma-related guilt - Trauma-related guilt inventory |
|  | Post Traumatic Growth - Posttraumatic Growth Inventory |
|  | Hopelessness - Beck Hopelessness Scale |
|  | Emotion regulation - The Abbreviated Dysregulation Inventory |
|  | Client Experience of Therapy |
|  | Client Change Interview |
|  | Personality - Minnesota Multiphasic Personality Inventory |
|  | Schemas - Young Schema Questionnaire |
|  | HONOS |
|  | Recidivism Risk - FARE |
|  | Coping - TCSQ |
|  | Distress - Outcome Questionnaire-45 Symptom Distress subscale |
|  | Borderline Personality Disorder Symptoms - Borderline Symptom List |
|  | Anger - Symptom Checklist - 90 revised Hostility Subscale |

| **4Section and Topic** | **Item #** | **Checklist item** | **Location where item is reported** |
| --- | --- | --- | --- |
| **TITLE** | | |  |
| Title | 1 | Identify the report as a systematic review. | Title page, p. 1. |
| **ABSTRACT** | | |  |
| Abstract | 2 | See the PRISMA 2020 for Abstracts checklist. | Abstract, pp. 2-3. |
| **INTRODUCTION** | | |  |
| Rationale | 3 | Describe the rationale for the review in the context of existing knowledge. | Introduction/Conceptualising Trauma/Drama-Based Strategies, pp. 4-7. |
| Objectives | 4 | Provide an explicit statement of the objective(s) or question(s) the review addresses. | Aim of this review, p. 8. |
| **METHODS** | | |  |
| Eligibility criteria | 5 | Specify the inclusion and exclusion criteria for the review and how studies were grouped for the syntheses. | Eligibility Criteria, pp. 9-10. |
| Information sources | 6 | Specify all databases, registers, websites, organisations, reference lists and other sources searched or consulted to identify studies. Specify the date when each source was last searched or consulted. | Search Strategy, p. 8; Supplementary Tables 1-2, Supp. pp. 1-4. |
| Search strategy | 7 | Present the full search strategies for all databases, registers and websites, including any filters and limits used. | Search Strategy, pp. 8-9; Supplementary Tables 1-2, Supp. pp. 1-4. |
| Selection process | 8 | Specify the methods used to decide whether a study met the inclusion criteria of the review, including how many reviewers screened each record and each report retrieved, whether they worked independently, and if applicable, details of automation tools used in the process. | Study Screening, pp. 9-10. |
| Data collection process | 9 | Specify the methods used to collect data from reports, including how many reviewers collected data from each report, whether they worked independently, any processes for obtaining or confirming data from study investigators, and if applicable, details of automation tools used in the process. | Data Extraction, p. 12. |
| Data items | 10a | List and define all outcomes for which data were sought. Specify whether all results that were compatible with each outcome domain in each study were sought (e.g. for all measures, time points, analyses), and if not, the methods used to decide which results to collect. | Eligibility Criteria (outcomes of interest), pp. 9-10; Data Extraction, p. 12. |
|  | 10b | List and define all other variables for which data were sought (e.g. participant and intervention characteristics, funding sources). Describe any assumptions made about any missing or unclear information. | Data Extraction, p. 12; Tables 1-2, pp. 16-20. |
| Study risk of bias assessment | 11 | Specify the methods used to assess risk of bias in the included studies, including details of the tool(s) used, how many reviewers assessed each study and whether they worked independently, and if applicable, details of automation tools used in the process. | Methodological quality assessment, pp. 12-13; Supplementary Table 4 and Supplementary Figures 1-5, Supp. pp. 5-12. |
| Effect measures | 12 | Specify for each outcome the effect measure(s) (e.g. risk ratio, mean difference) used in the synthesis or presentation of results. | Effect measures were inconsistently reported across studies; results were synthesised using direction of effect based on author-reported outcomes (Synthesis, pp. 13–14; Results, pp. 26–33). |
| Synthesis methods | 13a | Describe the processes used to decide which studies were eligible for each synthesis (e.g. tabulating the study intervention characteristics and comparing against the planned groups for each synthesis (item #5)). | Synthesis, pp. 13-14. |
|  | 13b | Describe any methods required to prepare the data for presentation or synthesis, such as handling of missing summary statistics, or data conversions. | Partially reported in Synthesis (pp. 13-14). |
|  | 13c | Describe any methods used to tabulate or visually display results of individual studies and syntheses. | Synthesis, pp. 13-14; Tables 1-3, pp. 16-23; Supplementary Tables 4-6, Supp. pp. 11-14. |
|  | 13d | Describe any methods used to synthesize results and provide a rationale for the choice(s). If meta-analysis was performed, describe the model(s), method(s) to identify the presence and extent of statistical heterogeneity, and software package(s) used. | Synthesis, pp. 13-14. |
|  | 13e | Describe any methods used to explore possible causes of heterogeneity among study results (e.g. subgroup analysis, meta-regression). | Not reported / not conducted. |
|  | 13f | Describe any sensitivity analyses conducted to assess robustness of the synthesized results. | Not reported / not conducted. |
| Reporting bias assessment | 14 | Describe any methods used to assess risk of bias due to missing results in a synthesis (arising from reporting biases). | Partially reported under Reporting Bias and Certainty of Evidence, p. 13; reporting bias later noted as not formally assessed, p. 25. |
| Certainty assessment | 15 | Describe any methods used to assess certainty (or confidence) in the body of evidence for an outcome. | Reporting Bias and Certainty of Evidence, p. 13; Supplementary Table 5, Supp. pp. 12-13. |
| **RESULTS** | | |  |
| Study selection | 16a | Describe the results of the search and selection process, from the number of records identified in the search to the number of studies included in the review, ideally using a flow diagram. | Study Screening, pp. 10-11; PRISMA flow diagram referenced in Figure 1 (text currently located in Methods rather than Results). |
|  | 16b | Cite studies that might appear to meet the inclusion criteria, but which were excluded, and explain why they were excluded. | NA |
| Study characteristics | 17 | Cite each included study and present its characteristics. | Study characteristics section, pp. 15-23; Tables 1-3, pp. 16-23. |
| Risk of bias in studies | 18 | Present assessments of risk of bias for each included study. | Critical appraisal of studies, pp. 24-25; Supplementary Figures 1-5 and Supplementary Table 4, Supp. pp. 5-12. |
| Results of individual studies | 19 | For all outcomes, present, for each study: (a) summary statistics for each group (where appropriate) and (b) an effect estimate and its precision (e.g. confidence/credible interval), ideally using structured tables or plots. | Narrative synthesis of individual study findings and outcome domains (Results, pp. 21–33; Table 3, pp. 22–23). Effect estimates and precision were not consistently reported in primary studies. |
| Results of syntheses | 20a | For each synthesis, briefly summarise the characteristics and risk of bias among contributing studies. | Critical appraisal of studies/Certainty of evidence, pp. 24-25, and narrative synthesis by intervention type, pp. 26-33. |
|  | 20b | Present results of all statistical syntheses conducted. If meta-analysis was done, present for each the summary estimate and its precision (e.g. confidence/credible interval) and measures of statistical heterogeneity. If comparing groups, describe the direction of the effect. | Narrative synthesis only; no meta-analysis undertaken. See pp. 13-14 and results pp. 26-33. |
|  | 20c | Present results of all investigations of possible causes of heterogeneity among study results. | Not reported / not conducted. |
|  | 20d | Present results of all sensitivity analyses conducted to assess the robustness of the synthesized results. | Not reported / not conducted. |
| Reporting biases | 21 | Present assessments of risk of bias due to missing results (arising from reporting biases) for each synthesis assessed. | Reporting bias not formally assessed, p. 25; see also Supplementary Table 5, Supp. pp. 12-13. |
| Certainty of evidence | 22 | Present assessments of certainty (or confidence) in the body of evidence for each outcome assessed. | Certainty of evidence, p. 25; Supplementary Table 5, Supp. pp. 12-13. |
| **DISCUSSION** | | |  |
| Discussion | 23a | Provide a general interpretation of the results in the context of other evidence. | Discussion, pp. 34-35. |
|  | 23b | Discuss any limitations of the evidence included in the review. | Discussion (limitations of included evidence), p. 36. |
|  | 23c | Discuss any limitations of the review processes used. | Discussion (limitations of the review process), pp. 36-37. |
|  | 23d | Discuss implications of the results for practice, policy, and future research. | Discussion (future research/implications) and Conclusion, pp. 37-38. |
| **OTHER INFORMATION** | | |  |
| Registration and protocol | 24a | Provide registration information for the review, including register name and registration number, or state that the review was not registered. | Search Strategy, p. 8 (PROSPERO ID: CRD420251165033). |
|  | 24b | Indicate where the review protocol can be accessed, or state that a protocol was not prepared. | Reported on p. 8 via PROSPERO registration number |
|  | 24c | Describe and explain any amendments to information provided at registration or in the protocol. | Not applicable. |
| Support | 25 | Describe sources of financial or non-financial support for the review, and the role of the funders or sponsors in the review. | Reported p.37 |
| Competing interests | 26 | Declare any competing interests of review authors. | Reported p.37 |
| Availability of data, code and other materials | 27 | Report which of the following are publicly available and where they can be found: template data collection forms; data extracted from included studies; data used for all analyses; analytic code; any other materials used in the review. | Reported p.37 |

*From:*  Page MJ, McKenzie JE, Bossuyt PM, Boutron I, Hoffmann TC, Mulrow CD, et al. The PRISMA 2020 statement: an updated guideline for reporting systematic reviews. BMJ 2021;372:n71. doi: 10.1136/bmj.n71. This work is licensed under CC BY 4.0. To view a copy of this license, visit <https://creativecommons.org/licenses/by/4.0/>
